# The Effectiveness of Electronic Health Interventions for Promoting HIV-Preventive Behaviors Among Men Who Have Sex With Men: Meta-Analysis Based on an Integrative Framework of Design and Implementation Features

**DOI:** 10.2196/15977

**Published:** 2020-05-25

**Authors:** Meiqi Xin, Kasisomayajula Viswanath, Angela Yuen-Chun Li, Wangnan CAO, Yuhong HU, Joseph Tak-Fai Lau, Phoenix Kit-Han Mo

**Affiliations:** 1 Jockey Club School of Public Health and Primary Care The Chinese University of Hong Kong Hong Kong China (Hong Kong); 2 TH Chan School of Public Health Harvard University Boston, MA United States; 3 Center for Evidence Synthesis in Health School of Public Health Brown University Providence, RI United States; 4 School of Public Health Shanghai University of Traditional Chinese Medicine Shanghai China

**Keywords:** HIV, AIDS, sexual and gender minorities, telemedicine, meta-analysis, systematic review

## Abstract

**Background:**

The disproportionately high prevalence of HIV among men who have sex with men (MSM) is a global concern. Despite the increasing utilization of electronic health (eHealth) technology in the delivery of HIV prevention interventions, few studies have systematically explored its effectiveness and association with various intervention characteristics.

**Objective:**

This study aimed to conduct a meta-analysis of the effectiveness of eHealth technology–based interventions for promoting HIV-preventive behaviors among MSM and to determine effectiveness predictors within a framework integrating design and implementation features.

**Methods:**

A systematic literature search using terms related to eHealth technology, HIV, the MSM population, and an experimental study design was performed using 5 databases (ie, MEDLINE, PsycINFO, EMBASE, Web of Science, and ProQuest Dissertations & Theses) and other sources (eg, bibliographies of relevant reviews and JMIR Publications). First, primary meta-analyses were conducted to estimate the effectiveness of eHealth interventions (*d*_+_) in changing 3 HIV-preventive behaviors among MSM: unprotected anal intercourse (UAI), HIV testing, and multiple sex partnership (MSP). Moderation analyses were then conducted to examine a priori effectiveness predictors including behavioral treatment components (eg, theory use, tailoring strategy use, navigation style, and treatment duration), eHealth technology components (eg, operation mode and modality type), and intervention adherence.

**Results:**

A total of 46 studies were included. The overall effect sizes at end point were small but significant for all outcomes (UAI: *d*_+_=−.21, *P*<.001; HIV testing: *d*_+_=.38, *P*<.001; MSP: *d*_+_=−.26, *P*=.02). The intervention effects on UAI were significantly larger when compared with preintervention groups than with concurrent groups. Greater UAI reductions were associated with the increased use of tailoring strategies, provision of feedback, and tunneling navigation in interventions with a concurrent group, whereas reductions were associated with the use of self-paced navigation in interventions with a preintervention group. Greater uptake of HIV testing was associated with longer treatment duration; computer-mediated communication; and the use of messaging, social media, or a combined technology modality. Higher intervention adherence consistently predicted larger effects on UAI and HIV testing.

**Conclusions:**

This study provided empirical evidence for the effectiveness of eHealth interventions in promoting HIV-preventive behaviors among MSM. Features of treatment content and eHealth technology might best predict the intervention effects on UAI and HIV testing, respectively. Most importantly, intervention adherence tended to play an important role in achieving better effectiveness. The findings could help inform the development of efficacious interventions for HIV prevention in the future.

## Introduction

### Background

In marked contrast to a declining trend in its global burden in the era of potent antiretroviral therapy, the HIV epidemic has continued to expand in the men who have sex with men (MSM) population across countries of all incomes in recent years [1,2]. The disproportionate burden is evidenced to be driven by a stable increase in HIV risk behaviors [3,4], which may offset the benefits of improved treatment coverage and biomedical advances [3,5]. Undiagnosed infections or infections diagnosed late arising from the relatively low uptake of HIV testing also fuel the epidemic by hampering treatment delivery and increasing transmission [6,7]. Therefore, the promotion of HIV-preventive behaviors is an indispensable part of comprehensive prevention efforts to control HIV [8]. Behavioral interventions among MSM have been found to significantly reduce risky sexual behaviors and increase HIV testing [9,10].

The global response to the ongoing epidemic warrants the application of innovative technology to develop efficacious behavioral interventions [7]. Electronic health (eHealth) is well recognized as the use of information and communication technologies for health, including websites, computerized programs or apps, social networking sites or chatrooms, email, or text messaging, that feature internet connectivity or digital interactivity via computer or mobile devices [11-13]. Previous quantitative reviews have shown that the impact of eHealth technology–based interventions on HIV prevention–related behaviors and theoretical correlates was significant but varied as a function of intervention characteristics [14,15].

New technology has been extensively used by MSM to socialize and seek sexual partners and to access information on sexual health [16,17]. An early meta-analysis revealed a significant effect of sexual health interventions delivered via interactive digital media for the overall population but not MSM, although a growing body of evidence has been accumulated since then [12]. Despite recent synthesis attempts focusing on MSM [18,19], the evaluation of effect magnitudes across outcomes and the disentanglement of efficacious components from complex intervention designs have been limited by the qualitative nature of the reviews. There is a lack of meta-analytic reviews that would allow rigorous testing of the a priori factors that predict intervention effectiveness [20] and thus inform the design and implementation of future programs.

### Potential Predictors of Electronic Health Intervention Effectiveness

An eHealth technology–based behavioral intervention includes 2 elements: *behavioral treatment* (what type of intervention is designed and programmed to target behavior change) and *eHealth technology* (how the treatment is delivered via eHealth platforms). This study proposed a conceptualized framework to capture critical factors that are theoretically and empirically demonstrated to explain the effectiveness of eHealth interventions.

#### Behavioral Treatment Components

Treatment content is characterized by the *use of theory* for intervention development, as theoretical constructs can be operationalized into treatment techniques [21]. Significantly greater reductions in unprotected anal intercourse (UAI) among MSM have been observed for interventions reporting any use of theory [9], yet it is unclear whether the extent of use predicts intervention effectiveness, particularly in the eHealth context. A meta-analysis of internet-based behavioral interventions across health domains revealed a significant association between more extensive theory use and increased effect sizes [22].

Treatment content also features the application of *tailoring strategies*, defined as the integration of recipients’ responses into the intervention system to generate user-driven content [23]. There are 3 tailoring types: (1) *feedback*: providing unique recommendations derived from an assessment of individual needs or characteristics related to a given behavior; (2) *adaptation*: matching content to a relevant group based on known behavioral determinants; and (3) *personalization*: customizing content with personally identifiable information [24]. Computer technology–based interventions with individually tailored content adapted to the stage of change were shown to be more efficacious in increasing condom use in the general population [14].

The programming features of a treatment can strongly determine the intervention intensity. The amount of accessible content per interaction with the intervention differs by *navigation style*: *tunneled* interventions deliver treatment through a predetermined sequence of steps, whereas *self-paced* interventions release content all at once and permit recipients to control the navigation [25-27]. Existing evidence is inconclusive regarding the influence of navigation style on the effectiveness of eHealth interventions [26,28,29]. *Treatment duration*, referring to the time span of delivery, represents the overall intervention burden. Larger effects and higher adherence have been generally reported for online behavioral interventions of a shorter duration [23].

#### Electronic Health Technology Components

On the basis of the nature of users’ interaction with eHealth platforms, 2 *operation modes* of technology use for intervention delivery have been distinguished: (1) *human-computer interaction (HCI)*, featuring direct interaction with a computerized system and automated delivery of a preprogrammed treatment and (2) *computer-mediated communication (CMC)*, featuring remote delivery through interpersonal communications via eHealth media. These are considered to be inseparable, albeit essentially different aspects of eHealth technology and capable of deploying similar treatment strategies [25,30]. It has been reported that the provision of remote therapeutic support, not fully automated treatment, achieved significantly better psychological outcomes than passive control [31].

Four *modality types* have been applied to eHealth interventions for HIV prevention, and each of them possesses a unique capacity to facilitate intervention delivery [11,19,32]. The 2 most common modalities are the *interactive module,* in which users actively engage in an intervention following a preset workflow, and the *static site,* in which users passively receive prescriptive information [30]. A previous review suggested the superiority of interactive over static interventions in predicting increased condom use and sexually transmitted infection (STI) testing [12]. The strength of *text messaging* lies in the ubiquitous use of mobile devices to deliver real-time personalized interventions [11,33]. It can incorporate effective communication techniques and has demonstrated its efficacy in promoting health behavior change [33,34]. This study uses the broader term, “messaging,” to encompass related technologies that enable multimedia delivery [33]. *Social media* has emerged as a novel modality that features the creation and sharing of user-generated content in an online community [32,35], which can effectively promote HIV testing [35].

#### Exposure to Intervention Components

Intervention effectiveness observed in real-world practice can change with actual exposure to efficacious intervention components, and nonusage attrition is common in eHealth interventions [36]. *Intervention adherence* is conceptualized as the proportion of participants who engage in the intervention as prescribed to achieve a desired effect [29]. This allows for a comparable measurement of intervention exposure across use parameters, by contrasting the *actual usage* of an intervention during its implementation with the *intended usage* predefined at the design stage [25]. Adherence has been found to significantly predict the effectiveness of eHealth interventions [23].

This study aimed to conduct a meta-analysis of the effectiveness of eHealth technology–based interventions for promoting HIV-preventive behaviors among MSM and to provide an in-depth investigation of effectiveness predictors.

## Methods

The guidelines of the Preferred Reporting Items for Systematic Reviews and Meta-Analyses were followed [37].

### Search and Selection

A systematic search was first performed in the following databases: MEDLINE, PsycINFO, EMBASE, Web of Science, and ProQuest Dissertations & Theses. The search strategy comprised 4 categories of terms related to eHealth technology, HIV or AIDS, the MSM population, and experimental study design, which were tailor-made for each database with restrictions to the English language and human studies (Multimedia Appendix 1). We manually searched bibliographies of relevant reviews, initially retrieved articles, and JMIR Publications to identify additional eligible studies. The eligibility criteria are listed in Table 1.

**Figure 1:**
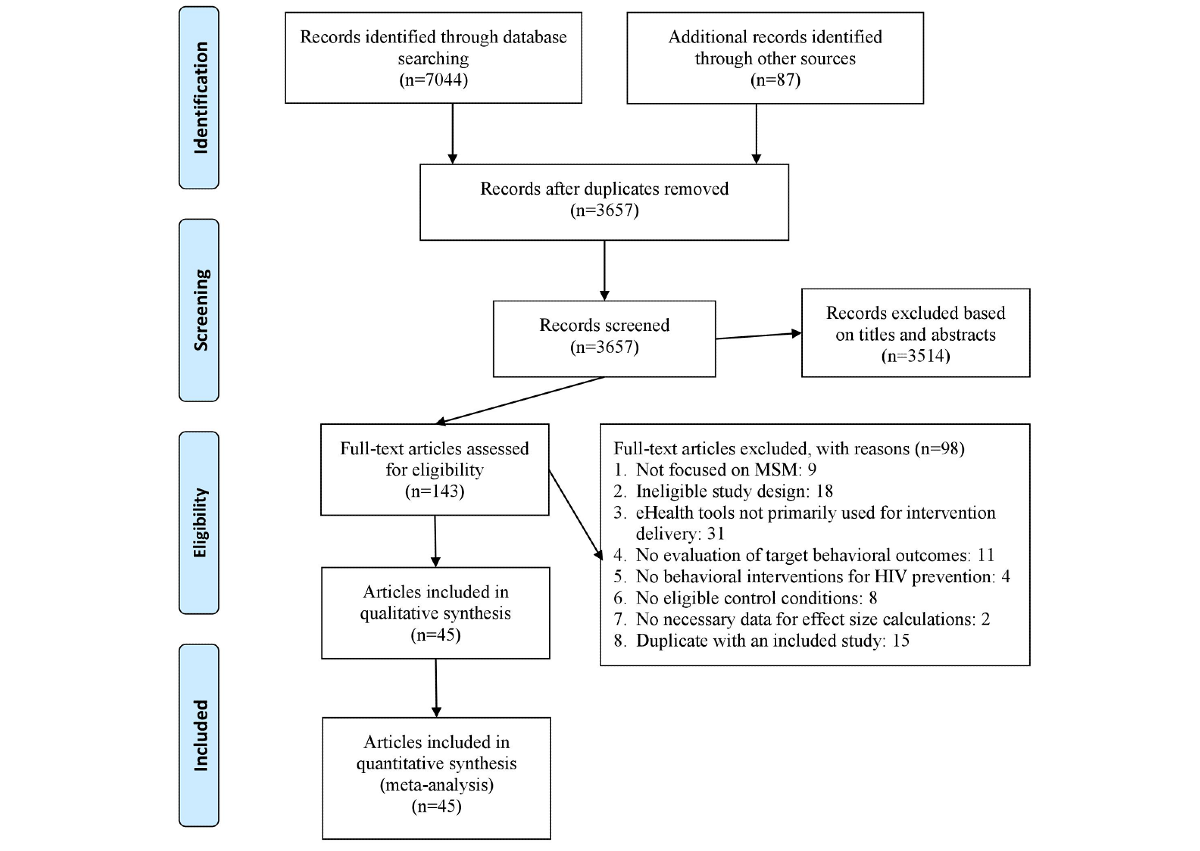
The Preferred Reporting Items for Systematic Reviews and Meta-Analyses flowchart of the screening process. eHealth: electronic health; MSM: men who have sex with men.

After removing the duplicates, 3657 relevant articles were identified from all the search sources. The title- and abstract-based eligibility screening was first conducted by 2 independent reviewers, which led to a selection of 143 articles for further full text–based assessment. Finally, 45 articles with a total sample size of 27,704 were deemed eligible and included in this meta-analysis [38-82]. The selection process is shown in Figure 1.

**Table 1:** Eligibility criteria for elements of a comprehensive search strategy.

Element	Inclusion and exclusion criteria
Population	Included: being exclusively or primarily (accounting for at least 80% of the sample) focused on MSM^a^ or specifically conducting the efficacy evaluation among the MSM subgroup
Intervention	Included: delivering interventions largely via eHealth^b^ technologies, including internet-based tools or interactive computerized programs and administering behavior change interventions for the prevention of HIV infection Excluded: solely using non-eHealth technologies (eg, telephone-based communications); not specifying the role of eHealth components in a multimedia program; or solely adopting biomedical strategies (eg, pre-exposure prophylaxis) or targeting the HIV care continuum after diagnosis (eg, treatment as prevention) or other sexual health topics (eg, contraception)
Comparator	Included: using control conditions that are different from interventions in the eHealth components Excluded: solely aimed at testing a group difference in other factors (eg, sample characteristics or treatment approaches)
Outcome	Included: measuring HIV-preventive behaviors as efficacy outcomes and providing necessary statistics to estimate the effect size Excluded: treating HIV-preventive behaviors as confounders or compensatory outcomes (eg, condom use measured in interventions on promoting monogamy); solely measuring behavioral correlates (eg, intentions) or biomarkers of engagement in HIV risk behaviors (eg, sexually transmitted infection occurrence); or computing a composite score for a group of behaviors
Study design	Included: a randomized controlled trial or nonrandomized experiment Excluded: a single-group posttest-only experiment; an observational study; or a review or commentary

^a^MSM: men who have sex with men.

^b^eHealth: electronic health.

### Quality Assessment

In all, 44 intervention programs were involved (2 articles reported outcome measures at different follow-up points for the same program). Program quality was evaluated using the assessment tool of Schnall et al [19], which was developed based on the reporting standards for HIV intervention studies established by the HIV Prevention Research Synthesis Team of the US Centers for Disease Control and Prevention [83] and has been well applied in previous reviews [9,19]. An overall percentage score (0%-100%) was computed as the extent to which each program fulfilled the criteria across 7 categories: representativeness, bias and confounding, description of intervention, outcomes and follow-up, statistical analysis, strength of evidence, and group equivalence.

### Data Extraction

#### Program Design

Data were extracted from each eligible program and its supplementary materials by one coder (MX) and double-checked by a second coder (AL). Information was first gathered on factors related to the program design, which were used to define the coding unit and to determine the grouping sets for the primary meta-analysis.

One intervention condition and one comparator were selected to form an independent study (a pairwise comparison) for the meta-analysis. If more than one intervention group was reported, each of them was separately included, given the potential variation in interventions, while the initial control group was proportionally divided up among intervention conditions to ensure independence [84]. If more than one control group was reported, the less intensive one was included for ease of interpretation [85]. If an eligible concurrent comparator was not obtained, or independent group contrasts were not the program focus, the preintervention status was treated as the comparator [86]. Finally, for each established comparison, identification items (eg, author) and sample characteristics (eg, age) were coded.

*Study design* was classified according to the outcome assessment points and comparison status (ie, using a concurrent or preintervention group). *Comparator type* was coded as (1) passive, including blank, waitlist, and attention controls or (2) active, including non-eHealth and lower tech eHealth treatment. *Follow-up period* was classified according to the interval between the intervention end and the follow-up points: (1) immediately postintervention; (2) short-term: ≤3 months; or (3) long-term follow-up: >3 months. The *outcome measures* with adequate studies for effect size pooling were UAI, condom use, HIV testing, and multiple sex partnership (MSP).

#### Intervention Features

A cluster of prespecified factors that captured the intervention characteristics were coded within the conceptualized framework described earlier. A relative coding rule was applied, that is, where a certain item was identical across the paired conditions, the study was coded as not having that feature, as it could not explain the conditional difference in the effectiveness.

With regard to the key features of treatment content, the extent of *theory use* was first assessed using the modified Theory Coding Scheme [21]. A total of 11 items pertinent to intervention development were used to compute an overall score [22], with a higher value indicating more extensive use of the theory. The second content feature was the *use of tailoring strategies*, which was coded in both ways, binary (whether adopting the *feedback*, *adaptation*, or *personalization* strategy) and continuous (the number of strategies adopted). The programming-specific treatment features were categorized into mutually exclusive groups: *navigation style* (self-paced or tunneled) and *treatment duration* (single session, ≤1 month, >1-3 months, and >3 months).

The utilization of eHealth technology was characterized by the *operation mode* (ie, HCI and CMC) and the *modality type* (ie, static site, interactive module, messaging, and social media). For both variables, interventions with the presence of more than one feature were grouped into a combined category to allow for exclusive coding. As indicators of intervention exposure, *intended usage* was first extracted and varying levels of *actual usage* were identified accordingly. *Intervention adherence* was then calculated as the percentage of participants whose actual usage matched the intended usage [25].

### Data Synthesis

#### Effect Size Calculation

Standardized mean differences between conditions (Cohen *d*) and their standard error at each follow-up point were derived to represent the magnitude of intervention effects [87,88]. Following Morris and DeShon’s [89] procedures, the effect size estimate and its sampling error were computed or transformed to be scaled on a common “raw-score” metric, creating a synthesis across the study design. Unadjusted outcome measurements were retrieved to establish comparability across estimates. For studies in which means and standard deviations were not provided, effect measures reported in other forms (eg, risk ratio) were converted to the Cohen *d* statistic using well-developed calculators [90,91].

As recommended, UAI and condom use were combined into one outcome type, “UAI” [14]. For studies that used multiple instruments to measure an outcome, the most common instrument was chosen (eg, the frequency of UAI was prioritized over the count of UAI partners [56]). For studies measuring different subtypes of a certain outcome on the same scale (eg, serostatus-specific UAI [71]) or reporting subgroup effects by significant moderators that were unrelated to intervention components (eg, affect level [44]), all effect sizes were aggregated meta-analytically within the study. Consequently, each study yielded only one Cohen *d* value per outcome.

#### Meta-Analysis

With an assumption of the intervention diversity, pooled effect sizes (*d*_+_) and 95% confidence intervals were generated using random effects models with the inverse variance weighting method. Values such as 0.2, 0.5, and 0.8 were interpreted as small, moderate, and large, respectively [92]. Primary meta-analyses were performed to combine effect sizes within different study sets for each outcome: (1) an overall estimate at the end point (one effect arising per study); (2) group-specific estimates of the end point effect by comparison status (nested groupings); and (3) group-specific estimates by follow-up period (a multi-wave study contributing multiple effects). To maximize analytical power, the first type of estimation based on a full data set was used for further analyses.

Heterogeneity across studies was assessed by using the Q statistic with a *P* value <.05, indicating the presence of significant heterogeneity, and quantified by using the I^2^ statistic, where a value of 30% to 60% denoted “moderate” and ≥75% denoted “considerable” heterogeneity [93]. Wherever considerable heterogeneity was found, outliers were identified as studies that significantly distorted the pooled effect using influence analyses [94] and were removed to ensure the accuracy and generalizability of the findings. Publication bias was then assessed by visually inspecting the funnel plot of effect sizes and conducting an Egger regression test to examine the plot asymmetry when there were at least 10 estimates [95].

Next, secondary analyses were performed to test the moderation effect of intervention features where adequate studies were available (n≥10) [96]. Subgroup and meta-regression analyses were applied for dichotomous and continuous measures, respectively; a significant moderator was indicated by a *P* value <.05 for the heterogeneity across subgroups (Q_b_) or the regression coefficient (β). All analyses were conducted in R 3.5.2 with the metafor package (Wolfgang Viechtbauer) [97].

## Results

### Descriptions of Program Characteristics

Over half of the eligible programs (23/44, 52%) were conducted in the United States, and 10 and 8 programs were conducted in Asia and Europe, respectively. Among programs reporting the respective background characteristics, most of the samples had a mean age below 30 years (24/44, 54%), were multiracial or Asian (29/39, 74%), were mainly (≥70%) composed of homosexual males or gays (22/28, 78%), and were non-HIV positive or of mixed (both positive and negative) status (33/36, 92%). In addition, the median overall quality score was 84.2%; the assessment results are provided in detail in Multimedia Appendix 2. A total of 46 studies were further identified from these programs; most of them conducted a concurrent comparison (n=29) and used a passive comparator (n=39). The study characteristics are provided in detail in Multimedia Appendix 3.

### Results of Primary Analyses

#### Unprotected Anal Intercourse

In combing all 35 studies that tested for UAI, a significant and small overall intervention effect was observed at end point (*d*_+_=−.32; *P*<.001), albeit with considerable heterogeneity (I^2^=83.3%). One study included subjects who reported recent condom-less sex with partners of either gender before enrollment; such a high-risk preintervention status might have contributed to the greatly decreased UAI (*d*=−1.13; SE 0.21) [49]. Moreover, the sample had the highest mean age of 45.1 years among all studies. Another study was focused on MSM sex workers and primarily addressed the context of transactional sex [63]. An extremely positive effect was found for reduced UAI with nonpaying male partners (*d*=−3.95; SE 0.35). Removing both outliers resulted in a smaller effect with moderate heterogeneity (*d*_+_=−.21; *P*<.001; I^2^=50.8%).

**Figure 2:**
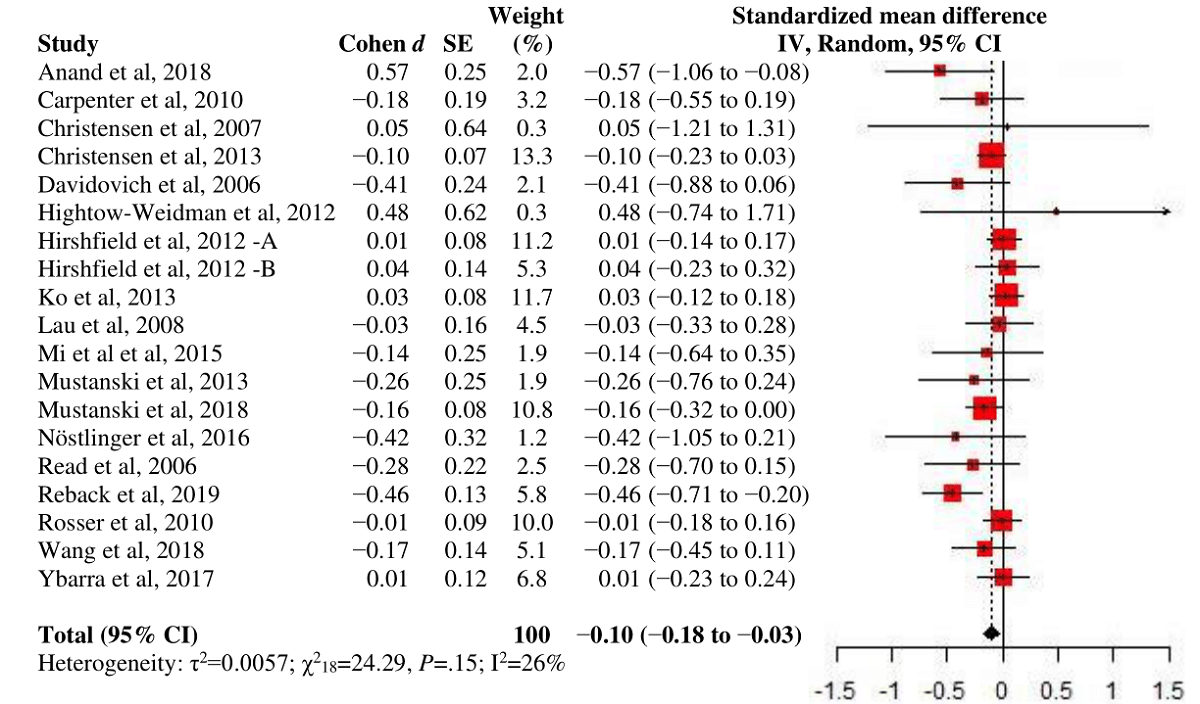
A forest plot of intervention effects on unprotected anal intercourse against concurrent comparison groups.

**Figure 3:**
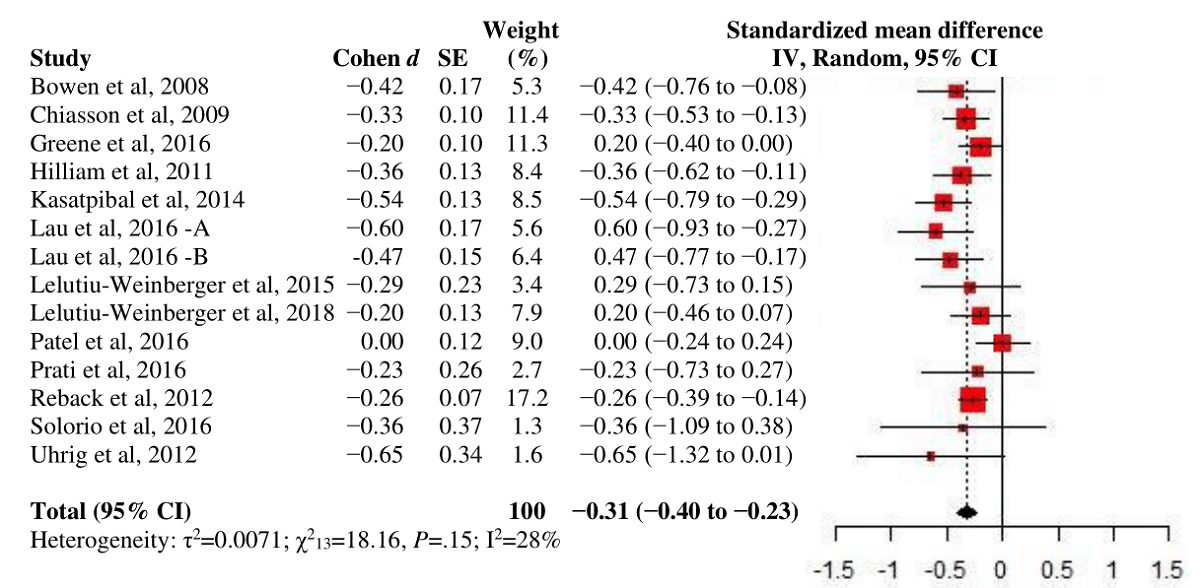
A forest plot of intervention effects on unprotected anal intercourse against preintervention comparison groups.

When stratified by the partner-specific outcome, a larger end point *d*_+_ was shown for UAI with main partners than with nonmain partners. When stratified by the follow-up period, a significant *d*_+_ was obtained at all points. The nested subgroup analysis found a significantly greater intervention effect when compared with preintervention groups than with concurrent groups (Q_b_=13.38; *P*<.001); hence, the meta-analysis was performed separately for the 2 types of comparison status as recommended (Figures 2 and 3) [89].

#### HIV Testing

**Figure 4:**
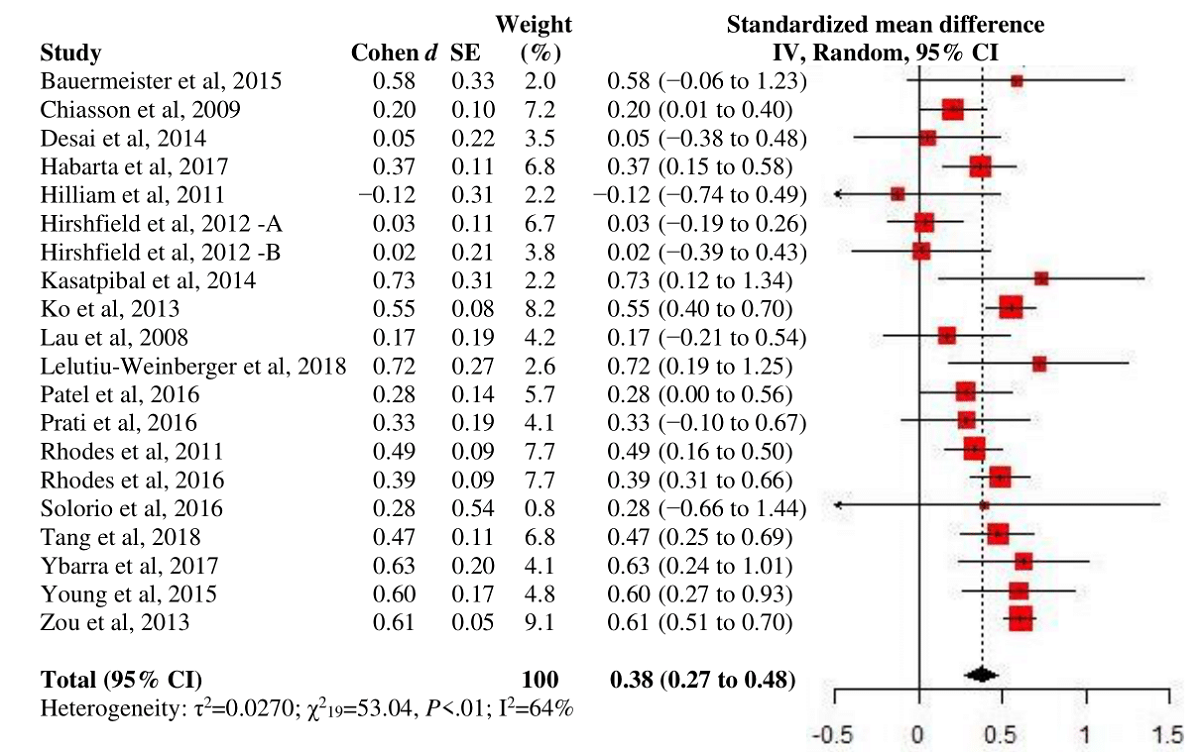
A forest plot of intervention effects on HIV testing.

Similarly, pooling the studies that tested for HIV testing revealed a significant, small overall effect at endpoint (n=23; *d*_+_=−.32; *P*<.001). The considerable heterogeneity (I^2^=84.0%) identified was largely attributable to the detection of outliers. Bourne et al [40] tested a texting reminder intervention against a blank control group involving participants who had refused such reminders; thus, the extreme effect size (*d*=.80; SE 0.06) could have been susceptible to volunteer bias. Wang et al [79] promoted a novel self-testing approach and distributed free home-based testing kits; the uptake of any type of testing was found to be considerably higher in the intervention group (*d*=1.18; SE 0.14). Mikolajczak et al [62] unexpectedly found a negative but nonsignificant intervention effect on HIV or STI testing relative to an active comparator (*d*=−.13; SE 0.12). The exclusion of outliers caused a slight change in the effectiveness but substantially reduced heterogeneity (*d*_+_=.38; *P*<.001; I^2^=64.2%; Figure 4). A larger *d*_+_ was further shown at postintervention than at short-term follow-up; however, only one sample was followed up for more than 3 months. No significant group difference by comparison status was observed.

#### Multiple Sex Partnership

**Figure 5:**
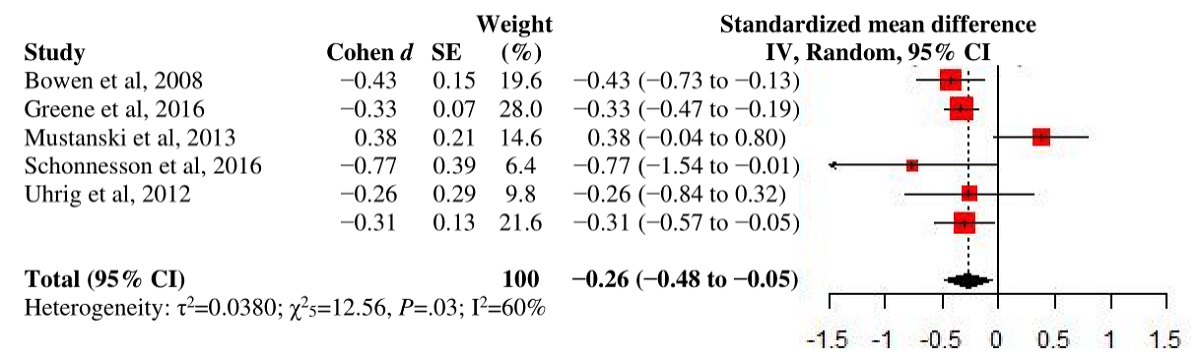
A forest plot of intervention effects on multiple sex partnership.

A significant, small overall effect was observed on the reduced MSP at endpoint with moderate heterogeneity (*d*_+_=−.26; *P*=.02; n=6; I^2^=60.2%; Figure 5). The largest *d_+_* was shown at medium-term follow-up among all follow-up periods, although only a few studies were available for each grouping. No between-group test was performed for comparison status because of the small number of studies. Publication bias was detected for none of the outcomes. More results of primary analyses are presented in Table 2.

**Table 2:** Overall effect sizes and stratification by study design features.

Studies			Population, n	Cohen *d* (95% CI)	*P* value	Q_w_ (*P* value)^a^	I^2^ (%)	Q_b_ (*P* value^b^)
**UAI^c^**								N/A^d^
	All studies at endpoint		35	−.32 (−.43 to −.20)	<.001	203.32 (<.001)	83.3	
	Studies at endpoint without outliers		33	−.21 (−.28 to −.14)	<.001	65.08 (<.001)	50.8	
	**Partner-specific UAI^e,f,g^**							N/A
		With main partners	6	−.42 (−.57 to −.27)	<.001	5.18 (.39)	3.4	
		With nonmain partners	9	−.20 (−.27 to −.13)	<.001	7.78 (.46)	0.0	
	**Follow-up period^f^**							N/A
		Postintervention	16	−.22 (−.36 to −.08)	.001	48.02 (<.001)	68.8	
		≤3 months^e^	19	−.21 (−.28 to −.12)	<.001	35.92 (.007)	49.9	
		>3 months^e^	6	−.26 (−.43 to −.08)	.004	11.60 (.04)	56.9	
	**Comparison status^e,g^**							13.38 (<.001)
		Concurrent	19	−.10 (−.18 to −.03)	.004	24.29 (.14)	25.9	
		Preintervention	14	−.31 (−.40 to −.23)	<.001	18.16 (.15)	28.4	
**HIV testing**								
	All studies at end point		23	.41 (.28 to .54)	<.001	137.43 (<.001)	84.0	
	Studies at end point without outliers		20	.38 (.27 to .48)	<.001	53.04 (<.001)	64.2	
	**Follow-up period^f^**							N/A
		Postintervention	15	.46 (.38 to .55)	<.001	23.49 (.05)	40.4	
		≤3 months^e^	8	.24 (.09 to .40)	.002	12.57 (.08)	44.3	
		>3 months	1	.47 (.25 to .69)	<.001	N/A	N/A	
	**Comparison status^e,g^**							0.42 (.52)
		Concurrent	11	.39 (.25 to .53)	<.001	36.67 (<.001)	72.7	
		Preintervention	9	.33 (.22 to .44)	<.001	9.14 (.33)	12.4	
**Multiple sex partnership**								
	All studies at endpoint		6	−.26 (−.48 to −.05)	.02	12.56 (.03)	60.2	
	**Follow-up period^f^**							N/A
		Postintervention	2	−.18 (−.33 to −.03)	.02	0.08 (.78)	0.0	
		≤3 months	3	−.19 (−.75 to .37)	.50	11.65 (.003)	82.8	
		>3 months	2	−.36 (−.56 to −.16)	<.001	0.34 (.56)	0.0	

^a^Q_w_ denotes the degree of within-group heterogeneity.

^b^Q_b_ denotes the degree of between-group difference in the pooled effect sizes.

^c^UAI: unprotected anal intercourse.

^d^N/A: not applicable.

^e^Studies with exclusion of outliers.

^f^Non-nested groupings: one study could contribute to more than one grouping.

^g^Effect sizes at the endpoint were pooled.

### Results of Moderation Analyses

#### Behavioral Treatment Components

Most of the studies were designed based on a theory (n=37); the most common one was the information-motivation-behavioral skills model (IMB; n=14). The intervention effects on UAI and HIV testing appeared to be comparable regardless of whether any theory was used. Among the theory-based studies, IMB was significantly associated with greater UAI reductions than other theories for concurrent comparisons (Q_b_=4.33; *P*=.04). The more extensive use of tailoring strategies was significantly associated with decreased UAI for concurrent comparisons (β=−.08; *P*=.01). Specifically, feedback was the only strategy that had a significantly negative effect on UAI (Q_b_=4.00; *P*=.04), whereas adopting the personalization strategy tended to have a positive effect on HIV testing (Q_b_=2.76; *P*=.10).

Significantly greater UAI reductions were shown for the tunneled (Q_b_=7.23; *P*=.01) and self-paced (Q_b_=4.23; *P*=.04) treatments relative to concurrent and preintervention groups, respectively. Such a moderating effect of navigation style was not detected for HIV testing. Longer treatments showed a significantly greater increase in HIV testing than those with a single session or those lasting ≤1 month (Q_b_=16.97; *P*<.001). However, the effects on UAI did not significantly differ across treatment durations.

#### Electronic Health Technology Components

Over half of the interventions were operated via HCI (n=26), and the others were in a CMC (n=10) or combined (n=8) mode. More studies used a single modality (n=32) than those incorporating multi-modalities in the same intervention albeit with varying combination patterns (n=14). No between-group difference was observed in the intervention effects on UAI by operation mode or modality type. The CMC and combined modes tended to present a larger *d_+_* value for HIV testing than did the HCI mode (Q_b_=2.76; *P*=.09). When separately exploring the effect of specific modes, the use of CMC predicted a significantly greater increase in HIV testing (Q_b_=4.38; *P*=.04), whereas HCI use showed no association with effectiveness. Moreover, a significantly larger *d_+_* value was found for the use of messaging, social media, and multi-modalities than for static sites (Q_b_=12.79; *P*=.005).

#### Intervention Adherence

Among all interventions, the adherence rates ranged from 25.5% to 100%. A higher adherence rate was significantly associated with a decrease in UAI for concurrent comparisons (β=−.27; *P*=.03). Given the highly negative skewed distribution, the studies were evenly divided into 3 levels to reflect the degree of relative adherence; the adherence rates were above 91.6% in the first tertile and below 76% in the third tertile. Intervention effects on HIV testing significantly differed by adherence level (Q_b_=7.28; *P*=.03). A moderate *d_+_* value was observed for high-level studies (in the first tertile) and a small one for mid- and low-level studies. Details of the intervention features are presented in Multimedia Appendix 4; more results of moderation analyses are presented in Multimedia Appendix 5.

## Discussion

### Principal Findings

This study identified 46 eligible studies published since 2006, which highlight the increasing application of eHealth technology in intervention delivery for HIV prevention over the last decade. Most studies tested the effectiveness of an entire intervention against a passive comparator. Those with an active comparator delivered a treatment that was either a basic component of a comprehensive intervention package [79] or existing online information that the intervention group might be exposed to as well [62]; hence, it is possible to isolate the effects of specific intervention components.

### Effectiveness of Electronic Health–Based Interventions

The primary meta-analysis consistently revealed a significant and small overall intervention effect for all the behavioral outcomes at endpoint. Considerable heterogeneity was nevertheless detected for UAI and HIV testing, and some influential cases were further identified to show extreme effect sizes and distinctive study characteristics. Removal of the outliers weakened the pooled effectiveness for both outcomes to some extent. In line with extant reviews, eHealth-based interventions exerted a greater impact on HIV testing [12] and the number of sex partners [14] than on condom use. Notably, the magnitude of effectiveness observed is seemingly higher than that in the general population [12], which suggests that the MSM community may benefit more than others from eHealth-based interventions.

Furthermore, the meta-analysis within the groupings by outcome type demonstrated greater reductions in UAI with main partners than with nonmain partners. It is recommended that future interventions target the partner-specific determinants of risky sexual behaviors. Most of the studies evaluated the postintervention and short-term effects across outcomes. Only 6 study samples were followed up for more than 3 months and showed a comparable decrease in UAI to that at earlier timepoints. It was not possible to estimate the long-term effect on HIV testing and MSP owing to the lack of data. This finding emphasizes the need to explore the effectiveness of eHealth interventions in maintaining behavior changes, especially given the necessity for consistent condom use and regular HIV testing [7,98]. The greater effect on UAI found for the pre-post comparison was probably confounded by factors unrelated to the intervention (eg, fatigue). Nevertheless, the heterogeneity between comparison types may also have resulted from the different intervention components deployed by the 2 groups of studies, as has been discussed in the following sections.

### Predictors of Intervention Effectiveness

#### Behavioral Treatment Components

This study confirmed the previous finding [14] that the use of the theory did not moderate the effectiveness of eHealth-based interventions for HIV prevention, although the majority of treatments were developed on a theoretical basis. The overall extent of theory use failed to significantly influence effectiveness across outcomes. However, this finding may merely reflect a lack of sensitivity in distinguishing an effect [99]. The number of tailoring strategies used significantly moderated effectiveness in reducing UAI, and only the most frequently used strategy, that is, feedback, was further shown to be effective. Treatments were less tailored for interventions promoting HIV testing, and only the moderating effect of personalization use reached marginal significance. Some evidence supporting the superiority of certain strategies has been reported [23]. The use of feedback and personalization commonly featured tailoring at an individual level, whereas the use of adaptation generated content matched to group-level factors.

Inconclusive results were found for the role of navigation style. Tunneled treatments achieved greater UAI reductions than self-paced treatments in studies using a concurrent group but lower reductions in those using a preintervention group. A plausible explanation is that the tunneling pattern differed across comparison types. A tunneled treatment by definition requires multiple interactions with the intervention, which could impair the effectiveness of eHealth behavioral interventions [23]. Over 60% of treatments with a preintervention group involved more than 5 modules, whereas only 24% of those with a concurrent group did so. Above all, the efficacy of the tunneling design itself is controversial as, on the one hand, the sequential release could ameliorate information anxiety and enhance the behavioral change process, whereas, on the other hand, the artificial confines could inhibit typical information-foraging behavior and intervention participants may lack the motivation to accommodate such constraints [100,101].

Longer treatments significantly predicted higher uptake of HIV testing but not reduced UAI, which adds to the evidence in favor of a difference in the influence of treatment duration on sexual and detection behaviors [12]. However, this finding might merely reject a linear relationship between treatment duration and the effect on UAI. A recent meta-analysis even demonstrated a negative effect of increased intervention length on intervention adherence and behavioral impacts, driven by decreased motivation over time [23].

#### Electronic Health Technology Components

Taking advantage of both human communication (eg, flexibility and rapport building [49]) and eHealth-enabled capacities (eg, convenience and anonymity [30]), the use of CMC was shown to be effective in increasing HIV testing. There was little evidence for the moderating effect of the operation mode on UAI, although only 1 intervention used a CMC mode. The results illustrate the need to incorporate human involvement into eHealth interventions. A variety of communicative functions have proved their efficacy in promoting behavior change, including counselor- or user-initiated conversations and peer-to-peer interactions [22]. Moreover, the greatest effectiveness was obtained with a combined mode, so the relevant question is not whether HCI or CMC is superior but, rather, how to combine them to maximize persuasiveness.

Similarly, intervention effects on the outcome of HIV testing but not UAI varied by modality type. Messaging, social media, and the combined type achieved a comparably small-to-medium effect. Messaging, largely operated via portable devices, affords individualized interventions (eg, location-based services) and dynamic connections (eg, response on request) [11,102]. Social media stands out for its capabilities to influence social norms and create a peer-supportive environment [103], especially within a closely connected community such as MSM [35]. It also enables interventions to blend into users’ daily lives by utilizing well-established platforms (eg, Facebook) [32]. The use of hybrid modalities may further generate a synergistic effect.

#### Intervention Adherence

Consistent with previous literature [23], a high degree of adherence to eHealth interventions predicted a protective effect on UAI and HIV testing. Assuming intention-to-treat as the motive to engage in an intervention, adherence is rooted in the properties of intervention design. Efficacious strategies (eg, self-monitoring), observable benefits (eg, health outcomes), and feasible programming (eg, appropriate workload) can all bolster engagement [26,36]. Usability of eHealth technology is also critical to minimizing discontinuance [36].

### Limitations

This study focused on the behavioral aspects of HIV prevention. Notably, it did not indicate the superiority of stand-alone behavioral interventions over other prevention tools (eg, pre-exposure prophylaxis) but was, rather, an attempt to explore the possibility of leveraging technological advances to strengthen an integral part of comprehensive biobehavioral prevention efforts, especially at a transition stage when the role of biomedical prevention is limited by its slow and uneven scale-up [7,104]. It is also impossible to synthesize evidence for other preventive behaviors owing to insufficient data (eg, serosorting) or highly heterogeneous measurements (eg, substance use). The potential interplay among intervention components has not been examined because of the lack of statistical power. Some other effectiveness predictors may also be missed (eg, eHealth literacy [26]), although the key factors with recognized terminology and accessible coding sources were selected. Finally, these findings could not represent studies reported in a language other than English, although there was no evidence of biased effectiveness among previous language-restrictive meta-analyses [105].

### Conclusions

This meta-analysis demonstrated eHealth technology to be a promising tool for delivering HIV prevention interventions among the MSM population. Nevertheless, there is limited evidence on the long-term impact of such interventions, and more research is warranted to investigate their application in non-Western contexts. Our findings suggested that enhanced behavioral treatment (eg, the use of tailoring strategies) determined the effect of eHealth interventions on UAI reductions, whereas appropriate programming (eg, longer treatment duration) and advanced eHealth technology (eg, the use of social media) predicted an increase in HIV testing. Future intervention design should focus on integrating efficacious treatment and technology components as well as on exploring their potential interplay. It is also recommended to incorporate implementation strategies to improve intervention adherence and, thus, achieve better effectiveness.

## Appendix

Multimedia Appendix 1 The search strategy.

Multimedia Appendix 2 Results of quality assessment.

Multimedia Appendix 3 Characteristics of 46 studies included in the present meta-analysis.

Multimedia Appendix 4 Design and implementation features of included interventions.

Multimedia Appendix 5 Results of moderation analyses.

## Abbreviations

CMC: computer-mediated communication
eHealth: electronic health
HCI: human-computer interaction
IMB: information-motivation-behavioral skills model
MSM: men who have sex with men
MSP: multiple sex partnership
STI: sexually transmitted infection
UAI: unprotected anal intercourse
