# Supplementary material for: The Effectiveness of Electronic Health Interventions for Promoting HIV-Preventive Behaviors Among Men Who Have Sex With Men: Meta-Analysis Based on an Integrative Framework of Design and Implementation Features
Source: J Med Internet Res. 2020 May 25;22(5):e15977. doi: 10.2196/15977 (PMC7281149; doi:10.2196/15977)
Supplement: Multimedia Appendix 1 [file jmir_v22i5e15977_app1.docx]

Multimedia Appendix 1. The search strategy.

Database 1: Ovid Medline

Search range: 1946 to Present (February 26, 2019) with Daily Update

| # | Search items |
| --- | --- |
| 1. eHealth Technology | |
| 1. | ehealth.mp. or exp Telemedicine/ |
| 2. | (Telemedicine or e-health or electronic health or mhealth or m-health or mobile health or telehealth).mp. [mp=title, abstract, original title, name of substance word, subject heading word, keyword heading word, protocol supplementary concept word, rare disease supplementary concept word, unique identifier, synonyms] |
| 3. | internet.mp. or exp Internet/ |
| 4. | (web or webs or website* or online).mp. |
| 5. | exp Video Games/ |
| 6. | (game* or gaming or gamification or videogame* or interactive video*).mp. |
| 7. | virtual reality.mp. |
| 8. | "social media".mp. or Social Media/ |
| 9. | exp Social Networking/ or social network*.mp. |
| 10. | (Facebook or Twitter or Grindr or Jack'd).mp. |
| 11. | forum*.mp. |
| 12. | (chat room* or chatroom*).mp. |
| 13. | exp Blogging/ or blog*.mp. |
| 14. | exp Text Messaging/ or text messag*.mp. |
| 15. | ("short message service" or SMS or instant messag*).mp. |
| 16. | email*.mp. or exp Electronic Mail/ |
| 17. | e-mail* or electronic mail*.mp. |
| 18. | exp Computers/ |
| 19. | computer*.mp. |
| 20. | (digital assistant* or PDA* or electronic tablet*).mp. |
| 21. | mobile device*.mp. |
| 22. | exp Cell Phones/ or mobile phone*.mp. |
| 23. | mobile telephone* or cell* phone* or cellphone* or cellular telephone*.mp. |
| 24. | (smartphone* or smart phone*).mp. |
| 25. | exp Mobile Applications/ or application*.mp. |
| 26. | (app or apps).mp. |
| 27. | 1 or 2 or 3 or 4 or 5 or 6 or 7 or 8 or 9 or 10 or 11 or 12 or 13 or 14 or 15 or 16 or 17 or 18 or 19 or 20 or 21 or 22 or 23 or 24 or 25 or 26 |
| 1. Health Topic | |
| 28. | exp HIV Infections/ or exp HIV/ |
| 29. | (HIV or AIDS or human immun* virus or acquired immun* syndrome).mp. |
| 30. | 28 or 29 |
| 1. Population | |
| 31. | bisexuality/ or homosexuality/ or exp homosexuality, male/ |
| 32. | (men who have sex with men or men who had sex with men or men having sex with men or MSM).mp. |
| 33. | ((gay or bisexual or homosexual or "same sex") and (male* or men)).mp. |
| 34. | 31 or 32 or 33 |
| 1. Study Design | |
| 35. | trial*.mp. or exp Clinical Trial/ |
| 36. | exp Human Experimentation/ |
| 37. | exp clinical trials as topic/ or exp controlled before-after studies/ or exp feasibility studies/ or exp pilot projects/ |
| 38. | (intervention* or experiment* or program* or project).mp. |
| 39. | exp Program Evaluation/ |
| 40. | (effect* or efficac* or effectiveness).mp. |
| 41. | 35 or 36 or 37 or 38 or 39 or 40 |
| 42. | 27 and 30 and 34 and 41 |
| 43. | limit 42 to (english language and humans) |

Database 2: PsycINFO

Search range: 1806 to February Week 1 2019

| # | Search items |
| --- | --- |
| 1. eHealth Technology | |
| 1. | exp Telemedicine/ or exp Electronic Communication/ or exp Online Therapy/ or ehealth.mp. |
| 2. | (telemedicine or telehealth or e-health or electronic health or mhealth or m-health or mobile health or telehealth).mp. [mp=title, abstract, heading word, table of contents, key concepts, original title, tests & measures] |
| 3. | internet.mp. or exp INTERNET/ |
| 4. | (web or webs or website* or online).mp. |
| 5. | exp computer games/ or exp simulation games/ |
| 6. | (game* or gaming or gamification or videogame* or interactive video*).mp. |
| 7. | exp Virtual Reality/ or virtual reality.mp. |
| 8. | exp Social Media/ or "social media".mp. |
| 9. | exp Social Networks/ or exp Online Social Networks/ or social network*.mp. |
| 10. | (Facebook or Twitter or Grindr or Jack'd).mp. |
| 11. | forum*.mp. |
| 12. | (chat room* or chatroom*).mp. |
| 13. | exp BLOG/ or blog*.mp. |
| 14. | exp Text Messaging/ |
| 15. | (text messag* or "short message service" or SMS or instant messag*).mp. |
| 16. | exp Computer Mediated Communication/ or email*.mp. |
| 17. | e-mail* or electronic mail*.mp. |
| 18. | exp computers/ |
| 19. | computer*.mp. |
| 20. | (digital assistant* or PDA* or electronic tablet*).mp. |
| 21. | exp Mobile Devices/ or mobile device*.mp. |
| 22. | exp Cellular Phones/ or mobile phone*.mp. |
| 23. | (cell* phone* or cellphone* or cellular telephone* or mobile telephone*).mp. |
| 24. | (smartphone* or smart phone*).mp. |
| 25. | (app or apps or application*).mp. |
| 26. | 1 or 2 or 3 or 4 or 5 or 6 or 7 or 8 or 9 or 10 or 11 or 12 or 13 or 14 or 15 or 16 or 17 or 18 or 19 or 20 or 21 or 22 or 23 or 24 or 25 |
| 1. Health Topic | |
| 27. | exp hiv/ or exp "aids (attitudes toward)"/ or exp aids prevention/ |
| 28. | (HIV or AIDS or human immun* virus or acquired immun* syndrome).mp. |
| 29. | 27 or 28 |
| 1. Population | |
| 30. | exp Same Sex Intercourse/ |
| 31. | homosexuality/ or exp male homosexuality/ or exp bisexuality/ |
| 32. | (men who have sex with men or men who had sex with men or men having sex with men or MSM).mp. |
| 33. | ((gay or bisexual or homosexual or "same sex ") and (male* or men)).mp. |
| 34. | 30 or 31 or 32 or 33 |
| 1. Study Design | |
| 35. | intervention*.mp. or exp INTERVENTION/ |
| 36. | experimental design/ or exp clinical trials/ or experimental methods/ or experimentation/ or exp quasi experimental methods/ |
| 37. | (experiment* or trial* or program* or project).mp. |
| 38. | exp program evaluation/ or exp treatment effectiveness evaluation/ |
| 39. | (effect* or efficac* or effectiveness).mp. |
| 40. | 35 or 36 or 37 or 38 or 39 |
| 41. | 26 and 29 and 34 and 40 |
| 42. | limit 42 to (human and english language) |

Database 3: Embase

Search range: 1910 to Present (February 26, 2019)

| # | Search items |
| --- | --- |
| 1. eHealth Technology | |
| 1. | exp Telemedicine/ or exp Electronic Communication/ or exp Online Therapy/ or ehealth.mp. |
| 2. | (telemedicine or telehealth or e-health or electronic health or mhealth or m-health or mobile health or telehealth).mp. [mp=title, abstract, heading word, table of contents, key concepts, original title, tests & measures] |
| 3. | internet.mp. or exp INTERNET/ |
| 4. | (web or webs or website* or online).mp. |
| 5. | exp computer games/ or exp simulation games/ |
| 6. | (game* or gaming or gamification or videogame* or interactive video*).mp. |
| 7. | exp Virtual Reality/ or virtual reality.mp. |
| 8. | exp Social Media/ or "social media".mp. |
| 9. | exp Social Networks/ or exp Online Social Networks/ or social network*.mp. |
| 10. | (Facebook or Twitter or Grindr or Jack'd).mp. |
| 11. | forum*.mp. |
| 12. | (chat room* or chatroom*).mp. |
| 13. | exp BLOG/ or blog*.mp. |
| 14. | exp Text Messaging/ |
| 15. | (text messag* or "short message service" or SMS or instant messag*).mp. |
| 16. | exp Computer Mediated Communication/ or email*.mp. |
| 17. | e-mail* or electronic mail*.mp. |
| 18. | exp computers/ |
| 19. | computer*.mp. |
| 20. | (digital assistant* or PDA* or electronic tablet*).mp. |
| 21. | exp Mobile Devices/ or mobile device*.mp. |
| 22. | exp Cellular Phones/ or mobile phone*.mp. |
| 23. | (cell* phone* or cellphone* or cellular telephone* or mobile telephone*).mp. |
| 24. | (smartphone* or smart phone*).mp. |
| 25. | (app or apps or application*).mp. |
| 26. | 1 or 2 or 3 or 4 or 5 or 6 or 7 or 8 or 9 or 10 or 11 or 12 or 13 or 14 or 15 or 16 or 17 or 18 or 19 or 20 or 21 or 22 or 23 or 24 or 25 |
| 1. Health Topic | |
| 27. | exp hiv/ or exp "aids (attitudes toward)"/ or exp aids prevention/ |
| 28. | (HIV or AIDS or human immun* virus or acquired immun* syndrome).mp. |
| 29. | 27 or 28 |
| 1. Population | |
| 30. | exp Same Sex Intercourse/ |
| 31. | homosexuality/ or exp male homosexuality/ or exp bisexuality/ |
| 32. | (men who have sex with men or men who had sex with men or men having sex with men or MSM).mp. |
| 33. | ((gay or bisexual or homosexual or "same sex ") and (male* or men)).mp. |
| 34. | 30 or 31 or 32 or 33 |
| 1. Study Design | |
| 35. | intervention*.mp. or exp INTERVENTION/ |
| 36. | experimental design/ or exp clinical trials/ or experimental methods/ or experimentation/ or exp quasi experimental methods/ |
| 37. | (experiment* or trial* or program* or project).mp. |
| 38. | exp program evaluation/ or exp treatment effectiveness evaluation/ |
| 39. | (effect* or efficac* or effectiveness).mp. |
| 40. | 35 or 36 or 37 or 38 or 39 |
| 41. | 26 and 29 and 34 and 40 |
| 42. | limit 42 to (human and english language) |

Database 4: Web of Science (core collection)

Search range: 1956 to Present (February 26, 2019)

| # | Search items |
| --- | --- |
| 1. eHealth Technology | |
| 1. | TOPIC: (ehealth OR e-health OR electronic health OR mhealth OR m-health OR mobile health OR telehealth OR telemedicine) |
| 2. | TOPIC: (internet OR web OR webs OR website* OR online) |
| 3. | TOPIC: (game* OR gaming OR gamification OR videogame* OR interactive video* OR virtual reality) |
| 4. | TOPIC: ("social media" OR social network* OR Facebook OR Twitter OR Grindr OR Jack'd) |
| 5. | TOPIC: (forum*) |
| 6. | TOPIC: (chat room* OR chatroom*) |
| 7. | TOPIC: (text messag* OR "short message service" OR SMS OR instant messag*) |
| 8. | TOPIC: (email* OR e-mail* OR electronic mail*) |
| 9. | TOPIC: (computer*) |
| 10. | TOPIC: (digital assistant* OR PDA* OR electronic tablet*) |
| 11. | TOPIC: (mobile device*) |
| 12. | TOPIC: (mobile phone* OR mobile telephone* OR cell* phone* OR cellphone* OR cellular telephone*) |
| 13. | TOPIC: (smart phone* OR smartphone*) |
| 14. | TOPIC: (application* OR app OR apps) |
| 15. | #14 OR #13 OR #12 OR #11 OR #10 OR #9 OR #8 OR #7 OR #6 OR #5 OR #4 OR #3 OR #2 OR #1 |
| 1. Health Topic | |
| 16. | TOPIC: (HIV OR AIDS OR human immun* virus OR acquired immun* syndrome) |
| 1. Population | |
| 17. | TOPIC: (men who have sex with men OR men who had sex with men OR men having sex with men OR MSM) |
| 18. | TOPIC: ((gay OR bisexual OR homosexual OR "same sex") AND (male* OR men)) |
| 19. | #18 OR #17 |
| 1. Study Design | |
| 20. | TOPIC: (intervention* OR trial* OR experiment* OR program* OR project) |
| 21. | TOPIC: (effect* OR efficac* OR effectiveness) |
| 22. | #21 OR #20 |
| 23. | #22 AND #19 AND #16 AND #15 |
| 24. | (#22 AND #19 AND #16 AND #15) AND LANGUAGE: (English) |

Database 5: ProQuest Dissertations & Theses

Search range: January 1, 1895 to Present (February 26, 2019)

| # | Search items |
| --- | --- |
| 1. eHealth Technology | |
| 1. | ti(ehealth OR e-health OR electronic health OR mhealth OR m-health OR mobile health OR telehealth OR telemedicine) OR ab (ehealth OR e-health OR electronic health OR mhealth OR m-health OR mobile health OR telehealth OR telemedicine) |
| 2. | ti (internet) OR ab (internet) |
| 3. | ti (web OR webs OR website* OR online) OR ab (web OR webs OR website* OR online) |
| 4. | ti (game* OR gaming OR gamification OR videogame* OR interactive video* OR virtual reality) OR ab (game* OR gaming OR gamification OR videogame* OR interactive video* OR virtual reality) |
| 5. | ti ("social media" OR social network* OR Facebook OR Twitter OR Grindr OR Jack'd) OR ab ("social media" OR social network* OR Facebook OR Twitter OR Grindr OR Jack'd) |
| 6. | ti (text messag* OR instant messag* OR "short message service" OR SMS) OR ab (text messag* OR instant messag* OR "short message service" OR SMS) |
| 7. | ti (email* OR e-mail* OR electronic mail*) OR ab (email* OR e-mail* OR electronic mail*) |
| 8. | ti (forum* OR chatroom* OR chat room* OR blog*) OR ab (forum* OR chatroom* OR chat room* OR blog*) |
| 9. | ti (computer* OR digital assistant* OR PDA* OR electronic tablet*) OR ab (computer* OR digital assistant* OR PDA* OR electronic tablet*) |
| 10. | ti (mobile device* OR mobile phone* OR mobile telephone* OR cell* phone* OR cellphone* OR cellular telephone*) OR ab (mobile device* OR mobile phone* OR mobile telephone* OR cell* phone* OR cellphone* OR cellular telephone*) |
| 11. | ti (smartphone* OR smart phone* OR application* OR apps OR app) OR ab (smartphone* OR smart phone* OR application* OR apps OR app) |
| 12. | 1 OR 2 OR 3 OR 4 OR 5 OR 6 OR 7 OR 8 OR 9 OR 10 OR 11 |
| 1. Health Topic | |
| 13. | ti (HIV OR AIDS OR human immun* virus OR acquired immun* syndrome) OR ab (HIV OR AIDS OR human immun* virus OR acquired immun* syndrome) |
| 1. Population | |
| 14. | ti ("men who have sex with men" OR "men who had sex with men" OR "men having sex with men" OR MSM) OR ab ("men who have sex with men" OR "men who had sex with men" OR "men having sex with men" OR MSM) |
| 15. | ti ((gay OR bisexual OR homosexual OR "same sex") AND (male* OR men)) OR ab ((gay OR bisexual OR homosexual OR "same sex") AND (male* OR men)) |
| 16. | 14 OR 15 |
| 1. Study Design | |
| 17. | ti (trial* OR intervention* OR experiment* OR program* OR project) OR ab (trial* OR intervention* OR experiment* OR program* OR project) |
| 18. | ti (effect* OR efficac* OR effectiveness) OR ab (effect* OR efficac* OR effectiveness) |
| 19. | 17 OR 18 |
| 23. | 12 AND 13 AND 16 AND 19 |
| 24. | (12 AND 13 AND 16 AND 19) AND (la.exact("ENG")) |
