# Supplementary material for: The Effectiveness of Electronic Health Interventions for Promoting HIV-Preventive Behaviors Among Men Who Have Sex With Men: Meta-Analysis Based on an Integrative Framework of Design and Implementation Features
Source: J Med Internet Res. 2020 May 25;22(5):e15977. doi: 10.2196/15977 (PMC7281149; doi:10.2196/15977)
Supplement: Multimedia Appendix 2 [file jmir_v22i5e15977_app2.docx]

Multimedia Appendix 2. Results of quality assessment.

| **Adequacy of Criteria (%) ^a^** | **1. Representa-tiveness** | **2. Bias and confounding** | **3. Description of intervention** | **4. Outcomes and follow-up** | **5. Statistical analysis** | **6. Strength of evidence** | **7. Group equivalence** | **Total score** |
| --- | --- | --- | --- | --- | --- | --- | --- | --- |
| Anand, 2018 | 75 | 75.0 | 66.7 | 50 | 100 | 100 | 33.3 | 71.4 |
| Bauermeister, 2015 | 100 | 91.7 | 100 | 100 | 100 | 50 | 100 | 91.7 |
| Bourne, 2011 | 75 | 75.0 | 33.3 | 50 | 100 | 100 | 50 | 69.0 |
| Bowen, 2008; Daniel, 2008 | 75 | 66.7 | 100 | 100 | 100 | 100 | 50 | 84.5 |
| Carpenter, 2010 | 75 | 66.7 | 66.7 | 75 | 100 | 100 | 50 | 76.2 |
| Chiasson, 2009 | 100 | 70.8 | 33.3 | 100 | 100 | 100 | 25 | 75.6 |
| Christensen, 2013 | 100 | 91.7 | 100 | 100 | 100 | 50 | 100 | 91.7 |
| Christensen, 2007 | 100 | 79.2 | 100 | 100 | 75 | 100 | 25 | 82.7 |
| Davidovich, 2006 | 100 | 70.8 | 100 | 100 | 100 | 100 | 100 | 95.8 |
| Desai, 2014 | 75 | 75.0 | 33.3 | 75 | 75 | 0 | 50 | 54.8 |
| Fernandez, 2016 | 100 | 79.2 | 100 | 100 | 100 | 100 | 100 | 97.0 |
| Greene, 2016 | 100 | 66.7 | 100 | 100 | 100 | 100 | 25 | 84.5 |
| Habarta, 2017 | 100 | 33.3 | 66.7 | 100 | 100 | 100 | 50 | 78.6 |
| Hightow-Weidman, 2012 | 100 | 79.2 | 100 | 100 | 100 | 0 | 25 | 72.0 |
| Hilliam, 2011 | 50 | 50.0 | 100 | 100 | 25 | 100 | 25 | 64.3 |
| Hirshfield, 2012 | 75 | 70.8 | 100 | 100 | 100 | 100 | 100 | 92.3 |
| Kasatpibal, 2014 | 50 | 87.5 | 100 | 50 | 100 | 100 | 75 | 80.4 |
| Ko, 2013 | 75 | 66.7 | 33.3 | 100 | 100 | 100 | 25 | 71.4 |
| Lau, 2008 | 75 | 79.2 | 66.7 | 100 | 100 | 0 | 25 | 63.7 |
| Lau, 2016 | 75 | 79.2 | 100 | 100 | 100 | 50 | 100 | 86.3 |
| Lelutiu-Weinberger, 2015 | 100 | 91.7 | 100 | 100 | 100 | 100 | 50 | 91.7 |
| Lelutiu-Weinberger, 2018 | 100 | 87.5 | 100 | 100 | 100 | 100 | 50 | 91.1 |
| Mi, 2015 | 75 | 87.5 | 33.3 | 100 | 100 | 50 | 25 | 67.3 |
| Mikolajczak, 2012 | 100 | 33.3 | 100 | 75 | 100 | 50 | 100 | 79.8 |
| Mimiaga, 2017 | 100 | 87.5 | 100 | 75 | 100 | 100 | 100 | 94.6 |
| Mustanski, 2013 | 100 | 100.0 | 100 | 100 | 100 | 100 | 100 | 100.0 |
| Mustanski, 2018 | 100 | 87.5 | 100 | 100 | 100 | 100 | 50 | 91.1 |
| Nöstlinger, 2016 | 75 | 79.2 | 100 | 100 | 100 | 100 | 100 | 93.5 |
| Patel, 2016 | 100 | 79.2 | 33.3 | 75 | 75 | 100 | 75 | 76.8 |
| Prati, 2016 | 25 | 66.7 | 66.7 | 100 | 100 | 100 | 75 | 76.2 |
| Read, 2006 | 75 | 66.7 | 100 | 100 | 100 | 100 | 50 | 84.5 |
| Reback, 2012 | 100 | 87.5 | 100 | 100 | 100 | 100 | 50 | 91.1 |
| Reback, 2019 | 100 | 87.5 | 100 | 75 | 100 | 100 | 50 | 87.5 |
| Rhodes, 2011 | 50 | 50.0 | 100 | 100 | 100 | 100 | 50 | 78.6 |
| Rhodes, 2016 | 50 | 66.7 | 100 | 100 | 100 | 100 | 50 | 81.0 |
| Rosser, 2010 | 100 | 87.5 | 66.7 | 75 | 100 | 0 | 100 | 75.6 |
| Schonnesson, 2016 | 75 | 66.7 | 100 | 75 | 100 | 100 | 75 | 84.5 |
| Solorio, 2016 | 100 | 87.5 | 66.7 | 100 | 100 | 50 | 25 | 75.6 |
| Tang, 2018 | 100 | 75.0 | 100 | 100 | 100 | 100 | 25 | 85.7 |
| Uhrig, 2012 | 100 | 87.5 | 100 | 100 | 100 | 100 | 25 | 87.5 |
| Wang, 2018 | 100 | 87.5 | 66.7 | 100 | 100 | 100 | 100 | 93.5 |
| Ybarra, 2017 | 75 | 62.5 | 100 | 75 | 100 | 100 | 75 | 83.9 |
| Young, 2015 | 100 | 87.5 | 100 | 75 | 100 | 100 | 100 | 94.6 |
| Zou, 2013 | 25 | 75.0 | 100 | 100 | 100 | 100 | 25 | 75.0 |

a. The quality assessment tool was developed by Schnall et al. [1] based on the reporting standards for HIV intervention studies established by the HIV Prevention Research Synthesis Team of U.S. Centers for Disease Control and Prevention [2, 3]:

1) Representativeness: refers to the descriptions of key characteristics of study population and of eligibility criteria;

2) Bias and confounding: refers to the generalizability to larger population, consistency in outcome assessment, controls of confounding interventions, and compliance rate;

3) Description of intervention: refers to the descriptions of intervention and/or monitoring;

4) Outcomes and follow-up: refers to the definition of outcome assessment procedure and group equivalence in attrition;

5) Statistical analysis: refers to the descriptions and appropriateness of statistical methods;

6) Strength of evidence: refers to the presence of significant positive intervention effects;

7) Group equivalence: refers to the use of appropriate comparison group(s), descriptions of group comparability and randomization method, and statistical controls of group inequivalence.

References:

1. Schnall R, Travers J, Rojas M, Carballo-Dieguez A. eHealth Interventions for HIV Prevention in High-Risk Men Who Have Sex with Men: A Systematic Review. J Med Internet Res 2014;16(5): 228-237. PMID: 24862459

2. Flores SA, Crepaz N, Synthesis HPR. Quality of study methods in individual- and group-level HIV intervention research: Critical reporting elements. AIDS Educ Prev 2004;16(4): 341-352. PMID: 15342336

3. Cynthia M. Lyles, Nicole Crepaz, Jeffrey H. Herbst, and Linda S. Kay. Evidence–Based HIV Behavioral Prevention from the Perspective of the CDC's HIV/AIDS Prevention Research Synthesis Team. AIDS Educ Prev 2006;18 (4 Suppl A): 21-31. PMID: 16987086
