# Supplementary material for: The Effectiveness of Electronic Health Interventions for Promoting HIV-Preventive Behaviors Among Men Who Have Sex With Men: Meta-Analysis Based on an Integrative Framework of Design and Implementation Features
Source: J Med Internet Res. 2020 May 25;22(5):e15977. doi: 10.2196/15977 (PMC7281149; doi:10.2196/15977)
Supplement: Multimedia Appendix 3 [file jmir_v22i5e15977_app3.docx]

Multimedia Appendix 3. Characteristics of 46 studies included in the present meta-analysis.

| **Study**  **/Location** | **Age (year)**  **/Race (Ethnicity)** | **Sexual orientation**  **/HIV status** | **Study design** **/Sample size** | **Intervention condition (INT)** | **Comparison condition (COM)** | **Follow-up period /Retention rate** | **Outcome type** |
| --- | --- | --- | --- | --- | --- | --- | --- |
| Anand, 2018 [1]  Thailand | Mean±SD: 28±5.9;  NR (presumed to be predominantly Asian) | NR;  Biologically tested: 100% (-) | IGPP: RCT  INT: 37  COM: 39 | COM plus 12 monthly HIV/STI prevention sessions delivered via Vialogues.com: using online time-based videos followed by time-stamped discussions with a health educator | Passive comparison: Standard of care;  Private clinic-based HIV counseling and testing at baseline, month 6 and 12 | Post-intervention:  88.2% | Condom use ^a^ |
| Bauermeister, 2015 [2]  *Go Connected!*  USA | Mean±SD: 21±2.23  65.6 % White, 19.5 % Black, 9.4 % Latino, 7.8 % Middle Eastern, and 6.3 % Asian/ Pacific Islander | 83.8% gay, 14.6% BIS, 1.6% HES, queer or same-gender loving;  Self-reported: 70.8% (-), 26.2% (+), 3.0% unknown | IGPP: RCT  INT: 86  COM: 44 | A tailored, personalized site: COM plus three web pages  of customized intervention content based on baseline psychosocial characteristics | Passive comparison:  Attention control  A non-tailored site: a web page of testing provider directory | 1 month:  80.0% | HIV/STI testing |
| Bourne, 2011 [3]  Australia | Mean (range): 33.6 (<25 – >40) ^b^;  NR | NR;  Self-reported:  100% (-) | IGPP: NRCT  INT: 714  COM: 2837 | SMS reminders for HIV/STI re-testing sent 4 months after the baseline test | Passive comparison:  Blank control;  Null | 5 months:  100.0% | HIV/STI retesting |
| Bowen, 2008 [4], Daniel, 2008 [5]  *WRAPP*  USA | Mean (range): 29.8 (18 – 80) ^b^;  78.1% Non-hispanic white, 9.1% Hispanic, 12.7% African American, Asian/ Pacific Islander, American Indian, other | 85% gay, 14.8% BIS, 0.3% HES;  NR | SGPP: Cohort  INT: 425  COM: 425 | An internet-based intervention that consisted of three modules (Knowledge, Partner, Contexts), each included two 20-min interactive sessions and printable feedback tailored to the participant's responses during the intervention | Passive comparison:  Pre-intervention status;  Null | Post-intervention: 69.2%  1- /9-months:  8.1% ^a^ | Condom use  MSP |
| Carpenter, 2010 [6]  USA | Mean±SD: 26.3±5.7;  15.2%Hispanic/Latin,6.3% African American,5.4% Asian American, 0.9% Hawaiian/ Pacific islander,7.1% Native American, 80.4% White, 2.7% Other (among follow-up completers) | NR;  Self-reported: 77.4% (-), 22.6% unknown | IGPP: RCT  INT: 99  COM: 100 | An internet-delivered multimedia safer sex intervention that consisted of seven motivational, informational and skills training modules with risk assessment and feedback, motivational exercises, skills training, and education | Passive comparison:  Attention control  A stress reduction training program | 3 months:  56.3% | UAI |
| Chiasson, 2009 [7]  USA | Mean (range): 30.1 (18 – >40) ^b^;  69.0% White, 6.1% Black, 13.7% Hispanic, 4.9% Asian and other, 2.3% Multi-race | 86.5% gay/HOS, 8.1% BIS, 5.4% other;  Self-reported: 13.8% (+); 86.2% unknown/ (-)  (among follow-up completers) | SGPP: Cohort  INT: 2707  COM: 2707 | The Morning After: Nine-minute video drama using professional actors and production team was created for online delivery to promote critical thinking about HIV risk | Passive comparison:  Pre-intervention status;  Null | 3 months:  19.3% | UAI  HIV testing  MSP |
| Christensen, 2013 [8]  *SOLVE-IT*  USA | Mean±SD: 21.3±1.8  13% Black/African American; 14% Hispanic/Latino; 73%White/Caucasian | 75.6% gay/HOS, 12.9% BIS, 11.0% other;  Self-reported: 100% (-) | IGPP: RCT  INT: 444  COM: 491 | A web-based SOLVE: a downable simulation video game featuring interactive narratives with intelligent characters that simulate and immerse MSM in affectively charged HIV risky situations | Passive comparison: Waitlist control;  Null | 3 months:  67.2% | UAI |
| Christensen, 2007 [9]  *SOLVE-IAV*  USA | Mean (range): 24.3 (18 – 30) ^b^;  47%White/Caucasian, 38% Hispanic/ Latino,  15% Black/ African-American | 84% gay/ HOS, 14% BIS, 2% other;  Self-reported: 100% (-) | IGPP: RCT  INT: 50  COM: 50 | A DVD-based SOLVE: three interactive HIV prevention videos with human actors across races, and players actively made choices for the main character, which foster associative learning | Active comparison:  Non-ehealth treatment;  Factually equivalent static videos: players passively watched scripted narratives | 3 months:  73.0% | UAI |
| Davidovich, 2006 [10]  The Netherlands | Mean±SD: 33±1.1;  21% non-Dutch, 79% Dutch | 63% exclusively attracted to men, 18% primarily attracted to men, 17% equally attracted to both sex, 2% primarily attracted to women;  Self-reported: 42%(-), 58% unknown | IGPP: RCT ^c^  INT: 340  COM: 333 | An online tailored intervention that consisted of information-providing, motivation-enhancing, and skill-building modules with intervention messages automatically compiled based on individual responses to a tailoring questionnaire | Passive comparison: Waitlist control;  Null | 6 months:  39.8% | UAI |
| Desai, 2014 [11]  UK | NR;  NR | NR;  Biologically tested: 100% (-) | IGPP: NRCT  INT: 31  COM: 656 | SMS reminders for HIV/STI re-testing offered at 3 months after the baseline test | Passive comparison:  Blank control;  Null | 2 months:  100.0% | HIV/STI retesting |
| Fernandez, 2016 [12]  *POWER*  USA | Mean±SD: 45.1±7.7;  93% African-American, 2 % Black-Latino, 3 % as Others, 2 % multiple  black identities  (among the entire program sample) | 84.3% BIS, 15.7% other  Self-reported: 34.6% (-), 65.4% (+) | SGPP: Cohort ^d^  INT: 84  COM: 108 | A real-time live-chat intervention delivered by facilitators on a dedicated website: three weekly sessions providing culturally relevant information on HIV risk and protection, and increasing motivation and behavioral skills to promote adoption of safe practices | Passive comparison:  Pre-intervention status;  Null | 3 months:  77.8% | UAI |
| Greene, 2016 [13]  *KIU! 1.5*  USA | Mean±SD: 21.5±1.9;  33.9% White, 31.6% African American, 21.6% Latino, 12.9% other | 73.4% HOS, 26.3% BIS/ other;  Biologically tested/ self-reported: 100% (-) | SGPP: Cohort  INT: 343  COM: 343 | An interactive online HIV prevention program involving seven modules completed across three sessions that use diverse delivery methods (e.g., videos, animation, and games) | Passive comparison:  Pre-intervention status;  Null | 3 months:  58.3% | UAI  MSP |
| Habarta, 2017 [14]  *TMUS*  USA | Mean (range): 29.2 (18 – 44) ^b^;  100% Black | NR;  Self-reported: 74.9% (-), 16% (+), 9.1% unknown | IGOP: CS  INT: 303  COM: 399 | A national multimedia campaign that used multiple digital channels: a dedicated website providing basic HIV/AIDS information, campaign information and a testing center search tool; and social media platforms | Passive comparison:  Blank control;  Null | Post-intervention:  NA | HIV testing  MSP |
| Hightow-Weidman, 2012 [15]  *HMP*  USA | Mean (range): 23.7 (19 – 30);  100% Black/ African-American | 63% gay, 31% BIS, 6% other;  Self-reported: 42% (+), 58% (-)/ unknown | IGPP: RCT  INT: 25  COM: 25 | An online HIV/STI prevention intervention via a dedicated interactive website featuring live chats with an HIV expert, interactive quizzes, personalized health and “hook-up/sex” journals, and decision support tools | Active comparison:  Lower tech-ehealth treatment;  Five currently available websites with general HIV/STI information | Post-intervention: 90.0%  2 months:  78.0% | Condom use |
| Hilliam, 2011 [16]  *HWUC*  UK | Mean (range): 37.1 (16 – >40) ^b^;  NR | NR;  NR | SGPP: RCS  INT: 775  COM: 88 | A national HIV prevention campaign delivered via digital online banners and targeted web pages, direct emails with key messages, and print materials | Passive comparison:  Pre-intervention status;  Null | Post-intervention:  NA | Condom use  HIV testing |
| Hirshfield, 2012 -A [17]  USA | Mean (range): 38.7 (18 – >50) ^b^;  82.1% White, 3.9% Black, 9.0% Hispanic, 5.0% Asian/ mixed/ other | NR;  Self-reported: 75.9% (-), 16.9% (+), 7.2% unknown | IGPP: RCT ^e^  INT-A: 1874  COM: 459 | INT-A: randomly assigned to one of three HIV prevention video conditions with the same story delivered in a dramatic or documentary or combined format to promote critical thinking | Passive comparison:  Placebo control;  Links to HIV prevention resources | 61 days:  48.7% | UAI  HIV testing |
| Hirshfield, 2012 -B [17]  USA | Mean (range): 37.6 (18 – >50) ^bb^;  78.8% White, 4.5% black, 8.8% Hispanic, 7.9% Asian/ mixed/ other | NR;  Self-reported: 74.2% (-), 18.2% (+), 7.5% unknown | IGPP: RCT ^e^  INT-B: 609  COM: 150 | INT-B: COM plus a CDC webpage that featured information about HIV among MSM | Passive comparison:  Placebo control;  Links to HIV prevention resources | 61 days:  46.8% | UAI  HIV testing |
| Kasatpibal, 2014 [18]  Thailand | Mean±SD: 23.7±6.0  NR (presumed to be predominantly Asian) | NR;  NR | SGPP: Cohort  INT: 162  COM: 162 | A dedicated website delivering instruction on HIV-prevention knowledge in the form of texts, pictures, animations, animated cartoons, videos, message boards, and exercises | Passive comparison:  Pre-intervention status;  Null | Post-intervention:  100.0% | Condom use  HIV/STI testing |
| Ko, 2013 [19]  *iPOL*  Taiwan | Mean±SD: 24.8±6.2  NR (presumed to be predominantly Asian) | NR;  Self-reported: 4.9% (+); 95.1% (-)/ unknown | IGPP: RCS  INT: 499  COM: 538 | The iPOLs actively disseminated HIV-related information via an online dedicated platform built on the Facebook social networking website, and discussed and responded to questions or replied to internet-using MSM | Passive comparison:  Blank control;  Null | Post-intervention:  NA | Condom use |
| Lau, 2008 [20]  Hong Kong | Mean (range): 26.1 (<20 – >41) ^b^;  (among follow-up completers)  99.3% Chinese, 0.7% non-Chinese | NR;  NR | IGPP: RCT  INT: 189  COM: 188 | INT-A: bi-weekly HIV information dissemination; a monthly electronic log-form for the self-monitoring of HIV risk behaviors and interactive tailored feedback via automatic emails | Passive comparison:  Placebo control;  Information pamphlets distributed during the recruitment exercise | Post-intervention:  74.3% | Condom use  HIV testing |
| Lau, 2016 -A [21]  Hong Kong | Mean (range): 27.1 (18 – > 41) ^b^;  100% Asian ^a^ | 73.6% HOS, 21.7% BIS, 0.8% HES, 3.9% unsure;  NR | SGPP: Cohort ^d^  INT: 133  COM: 133 | INT-A: two STI-related cognitions involving online videos: 1) a doctor presented STI information and prevention means; 2) peer MSM discussed practical means to prevent UAI | Passive comparison:  Pre-intervention status;  Null | 3 months:  70.7% | UAI |
| Lau, 2016 -B [21]  Hong Kong | Mean (range): 25.7 (18 – > 41) ^b^;  100% Asian ^a^ | 73.5% HOS, 21.2% BIS, 0% HES, 5.3% unsure;  NR | SGPP: Cohort ^d^  INT: 133  COM: 133 | INT-B: INT-A plus one more online video- a movie involving additional elements of fear appeal and visual imagery | Passive comparison:  Pre-intervention status;  Null | 3 months:  82.00% | UAI |
| Lelutiu-Weinberger, 2015 [22]  *MiCHAT*  USA | Mean±SD: 25.2±3.2;  USA: 53.7% White, 17.1% Black, 22% Latino, 7.3% Other | 85.4% gay, 12.2% BIS, 2.4% uncertain;  Self-reported: 100% (-) or unknown | SGPP: Cohort  INT: 27  COM: 41 | Eight weekly motivational interviewing and cognitive behavioral skills training -based live chat HIV prevention intervention sessions delivered on Facebook | Passive comparison:  Pre-intervention status;  Null | Post-intervention:  65.8% | UAI |
| Lelutiu-Weinberger, 2018 [23]  *DMDN*  Romania | Mean±SD: 23.4±3.6 (among follow-up completers);  100% Romanian | 70% gay, 28% BIS, 2% unsure (among follow-up completers);  Self-reported: 100% (-) or unknown | SGPP: Cohort  INT: 49  COM: 49 | Eight weekly motivational interviewing and cognitive behavioral skills training -based live chat HIV prevention intervention sessions delivered on a mobile site | Passive comparison:  Pre-intervention status;  Null | Post-intervention:  87.8% | UAI  condom use |
| Mi, 2015 [24]  China | Mean±SD: 30.9±8.2;  NR (presumed to be predominantly Asian) | NR;  Biologically tested: 100% (+) | IGPP: NRCT  INT: 104  COM: 98 | COM plus a web-based program with four modules: an information exchange website, a bulletin board system, individualized online counseling with trained peer educators, and an animation game | Passive comparison: Standard of care;  Clinic-based regular HIV care | Post-intervention:  99.0% | Condom use |
| Mikolajczak, 2012 [25]  *Queermasters*  The Netherlands | Mean: 36 (SD not available);  86.2% Dutch, 6.7% Dutch and other, 7.1% Non-Dutch | 86.9% HOS, 13.1% BIS;  Self-reported: 100% (-) | IGPP: RCT  INT: 870  COM: 834 | A dedicated website included multiple program components, virtual relational agents and virtual MSM audience, to introduce the regular Sexual Health Checkups, focusing on their advantages and attempting to influence the perceived social norm [26] | Active comparison:  Lower tech-ehealth treatment;  An existing online intervention that focused on both risk information and risk communication about HIV/STI testing | 3 months:  31.0% | HIV/STI testing |
| Mimiaga, 2017 [27]  India | Mean±SD: 27.7±9.1;  NR (presumed to be predominantly Asian) | NR;  Biologically tested: 3% (+), 97% (-) | IGPP: RCT  INT: 50  COM: 50 | COM plus an intervention integrating in-person and mobile phone delivered one-on-one HIV risk reduction counseling; and daily, personalized mobile messages as motivating “cognitive restructuring” cues | Passive comparison: Standard of care;  In-person HIV testing and counseling at baseline and month 6 | 3 months:  98.0%  6 months  98.0% | UAI |
| Mustanski, 2013 [28]  *KIU! 1.0*  USA | Mean±SD: 21.3±1.9  46.1% White-latino, 25.5% White-non-latino, 12.7% African American, 15.7% Native American, Asian, Mixed Race | 82.3% gay/HOS, 17.7% BIS/other;  Biologically tested: 100% (-) | IGPP: RCT  INT: 50  COM: 52 | An interactive online HIV prevention program involving seven modules completed across three sessions that use diverse delivery methods (e.g., videos, animation, and games); and a booster session for reinforcement at week 6 | Active comparison:  Lower tech-ehealth treatment;  An online program with 3 sessions of didactic, non-tailored and non-interactive HIV/STI facts (existing online) | 3 months:  88.2% | UAI  MSP |
| Mustanski, 2018 [29]  *KIU! 2.0*  USA | Mean (range): 23.8 (18 – 29) ^b^;  36.6% White, 24.3% Black, 28.9% Hispanic/ Latino, 10.2% other | 86.2% gay, 11.5% BIS, 2.2% straight/ other;  Biologically tested: 100% (-) | IGPP: RCT  INT: 445  COM: 456 | An interactive online HIV prevention program involving seven modules completed across three sessions that use diverse delivery methods (e.g., videos, animation, and games); and two booster sessions for reinforcement at 3- and 6-month | Active comparison:  Lower tech-ehealth treatment;  An online program with 3 sessions of didactic, non-tailored and non-interactive HIV/STI facts (existing online) | 3 months:  86.2%  6 months:  81.2%  12 months:  85.6% | UAI [30] |
| Nöstlinger, 2016 [31]  *CISS*  Europe | Mean±SD: 41.0±10.1;  NR | NR;  Biologically tested: 100% (+) | IGPP: RCT  INT: 55  COM: 57 | COM plus three individual counseling sessions facilitated by service providers, who guided participants in working through a series of video materials and interactive slide shows available on a DVD | Passive comparison: Standard of care;  Clinic-based sexual health counseling as regular HIV care; and an information leaflet on local sexual health services | 3 months:  67.00%  6 months:  67.9% | Condom use |
| Patel, 2016 [32]  *CHALO!*  India | Mean (range): 29.0 (18 – >42) ^b^;  NR (presumed to be predominantly Asian)  [33] | 71.5% HOS, 25.5% BIS, 3% HES;  Self-reported: 100% (-) [33] | SGPP: Cohort ^f^  INT: 130  COM: 244  [33] | Approach- or avoidance-framed messages delivered by peer educators via private Facebook group, individual WhatsApp messaging or email; and embedded links to an informational website on HIV prevention services [33] | Passive comparison:  Pre-intervention status;  Null | Post-intervention:  53.3%  [33] | Condom use  HIV testing  [33] |
| Prati, 2016 [34]  *United Against AIDS*  Italy | Mean±SD: 34.8±10.7;  NR | NR;  NR | SGPP: Cohort  INT: 199  COM: 282 | A national HIV/AIDS prevention campaign delivered via multiple media including Web-based advertisements, television and radio public service announcements, print materials, and cinema and newspaper advertisements | Passive comparison:  Pre-intervention status;  Null | Post-intervention:  70.6% | UAI  HIV testing |
| Read, 2006 [35]  *SOLVE-IAV*  USA | Mean (range): 33.5 (20 – 56) ^b^;  61.3% European American, 22.3% Latino/Hispanic, 8.7% African American/ Black, 3.9%Asian American, 1% Native American, 2.8% other | NR;  Biologically tested: 100% (-) | IGPP: RCT  INT: 88  COM: 48 | COM plus a DVD-based SOLVE video placing MSM in an interactive virtual environment designed to simulate the narrative of an actual sexual encounter while challenging and changing their more automatic patterns of risky responses | Passive comparison: Standard of care;  Standard one-on-one HIV peer-counseling at baseline | 3 months:  76.5% | Condom use [36] |
| Reback, 2012 [37]  *Project Tech Support*  USA | Mean±SD: 36.5±8.9;  34.6% Caucasian/ white, 21.2% African American/Black, 38.5% Hispanic/ Latino; 5.8% Multi/other | 80.8% gay; 19.2% BIS  Biologically tested: 59.6% (+), 40.4% (-) | SGPP: Cohort  INT: 52  COM: 52 | Daily social support and health education text messages transmitted in real-time by peer educators via mobile phone to reduce methamphetamine use and high-risk sexual behaviors | Passive comparison:  Pre-intervention status;  Null | 2 months:  92.3% | UAI |
| Reback, 2019 [38]  *Project Tech Support 2*  USA | Mean±SD: 41.7±10.8;  21.4% Caucasian/ White, 42.8% African American/ Black, 23.0% Hispanic/ Latino, 12.8% Multi/ other | 66.3% gay, 33.7% non-gay  Self-report: 40.6% (+), 59.4% (-) | IGPP: RCT ^c^  INT: 94  COM: 93 | COM plus interactive real-time text‑messaging conversations with peer health educators; and five-times-a-day theory‑based, gay‑specific text messages transmitted by automation to reduce methamphetamine use and high-risk sexual behaviors | Passive comparison:  Attention control  Weekly self-monitoring text-based assessment automatically delivered via mobile phone | Post-intervention:  82.9%  3 months:  90.4%  6 months:  84.0%  9 months:  90.9% | UAI |
| Rhodes, 2011 [39]  *CyBER/testing*  USA | Mean±SD: 37.1±11.5;  71.3%White,3%Black/African American, 1.8% Latino, 0.6% Asian/ Pacific Islander, 2.4% Mixed, 20.8% undisclosed  (among chatters at the study site) | 58% gay, 17.8% BIS, 24.2% undisclosed;  Self-reported:  1.6% (+); 22.6% undisclosed; 75.8% other  (among chatters at the study site) | SGPP: RCS  INT: 346  COM: 315 | A real-time interactive intervention on HIV testing delivered by a peer educator in a public chat room designed for MSM social and sexual networking and through its embedded private instant messaging | Passive comparison:  Pre-intervention status;  Null | Post-intervention:  NA | HIV testing |
| Rhodes, 2016 [40]  *CyBER/testing*  USA | Mean±SD:39.3±12.6^b^;  1.4% American Indian, 14.9% African American/ Black, 0.5% Asian/ Pacific islander, 6.3% Native Hawaiian, 1.8% Latino, 75.2% White | 45.3% gay, 41.0% BIS, 12.7% straight, 0.9% other;  Self-report: 10.7% (+); 89.3% (-)/ no past 12-month HIV testing | IGPP: RCS  INT: 339  COM: 314 | User-initiated communication on HIV testing with a peer educator through instant messaging or email within four existing social media sites designed for MSM social and sexual networking | Passive comparison:  Blank control;  Null | Post-intervention:  NA | HIV testing |
| Rosser, 2010 [41]  *MINTS-II*  USA | Mean (range) :34.0 (18 - > 45) ^b^;  68.2% White,6.3% African American/ Black, 15.1% Latino/ Spanish/other, 3.5% Asian, 6.9% Other | 91.4% HOS/gay/same gender loving, 8.6% BIS/ straight/other;  Self-report: 21.6% (+), 78.4% (-) | IGPP: RCT  INT: 337  COM: 313 | An internet-based HIV prevention intervention, *Sexpulse,* that included multiple highly interactive modules aimed to build a personal ‘portrait of sexual health,’ with each module yielding a portrait piece | Passive comparison: Waitlist control;  Null | 3 months:  86.2%  12 months:  85.2% | UAI |
| Schonnesson, 2016 [42]  *SMART*  Sweden | Mean±SD: 32±12.1;  NR | 93% gay, 7% non-gay  Self-reported  62% (-), 36% unknown, 1.7% (+) | IGPP: RCT  INT: 58  COM: 54 | An adaptation from the WRAPP to the Swedish context, with the intervention content left unaltered | Passive comparison: Waitlist control;  Null | 1 month:  51.8% | MSP |
| Solorio, 2016 [43]  *Tu Amigo Pepe*  USA | Mean±SD: 25±3.0;  100% Latino | 69.4% HOS, 20.4% BIS/ others, 10.2% HES;  Self-reported  100% (-)/ unknown | SGPP: Cohort  INT: 50  COM: 50 | A multimedia HIV testing campaign: a dedicated website, social media messaging, a mobile reminder system, radio PSAs, print materials, a hotline, in-person testing instructions, and the offer of free home-based self-testing kits | Passive comparison:  Pre-intervention status;  Null | Post-intervention:  88.0%  2 months:  82.0% | Condom use  HIV testing |
| Tang, 2018 [44]  China | Mean (range): 24.8 (16 – >30) ^b^;  NR (presumed to be predominantly Asian) | 68.5% gay; 31.5% BIS  Self-reported: 100% (-)/ unknown | SGPP: Cohort  INT: 824  COM: 824 | COM plus exceptional images about HIV testing disseminated via WeChat biweekly; an online HIV self-testing platform on WeChat; and local contests eliciting stories about HIV testing with finalists disseminated via social media | Passive comparison:  Pre-intervention status;  Routine promotional efforts by local Centers for Disease Control and community-based organizations | Post-intervention:  93.3%  3 months:  88.6%  6 months:  83.5% | HIV testing |
| Uhrig, 2012 [45]  USA | Mean (range): 38.4 (25 - >60) ^b^;  33% Black, 44%  White, 23% Other | 87% gay/HOS, 10% BIS, 4% Queer;  Biologically tested : 100% (+) | SGPP: Cohort  INT: 52  COM: 52 | A tailored SMS-based intervention delivered via mobile phones to reduce risk-taking behaviors and enhance HIV knowledge, social support, and patient involvement | Passive comparison:  Pre-intervention status;  Null | Post-intervention:  88.5% | UAI  MSP |
| Wang, 2018 [46]  Hong Kong | Mean (range): 29.0 (18- >40) ^b^;  100% Asian | 89.3% gay; 10.7% BIS  Self-reported: 100% (-)/ unknown | IGPP: RCT  INT: 215  COM: 215 | COM plus a home-based HIV self-testing service including mailing a free testing kit and providing online real-time instructions and pre-test/post-test counseling via online live-chat applications | Active comparison:  Lower tech-ehealth treatment;  A 3-min online video with general testing promotion | 6 months:  91.6% | UAI  HIV testing MSP |
| Ybarra, 2017 [47]  *Guy2Guy*  USA | Mean±SD: 16.1±1.6;  67.1% White, 14.8% African American, 18.0% All other races | 71.7% gay; 28.3% bisexual, and/or queer;  NR | IGPP: RCT  INT: 150  COM: 152 | Mobile phone-based text messaging: daily information, motivation and behavior messages reinforced by a booster; weekly gamelike messages; on-demand Q&A; and buddy suggestion text | Passive comparison:  Attention control  Attention-matched messages on general health topics | Post-intervention:  93.7%  3 months:  95.7% | UAI  HIV testing |
| Young, 2015 [48]  *HOPE*  Peru | Mean±SD: 28.9±7.9;  19.6% White, 2.3% Black, 69.8% Mixed, 8.3% other | 76.3% HOS; 19.1% BIS; 4.7% other;  Self-reported: 100% (-)/ unknown | IGPP: RCT  INT: 278  COM: 278 | COM plus peer leader-led communications with assigned participants about HIV prevention and testing on Facebook by sending messages, chats and wall posts in the private groups | Active comparison:  Lower tech-ehealth treatment;  Standard prevention and testing services; testing information via private Facebook groups; an offer of a free HIV test | Post-intervention:  89.6% | HIV testing |
| Zou, 2013 [49]  Australia | Mean: 30.3 ^b^;  (98.3% of the sample)  NR | NR;  NR | IGPP: NRCT  INT: 997  COM: 1382 | Reminders for STI screening using automated text messages sent to mobile telephones or/and emails every 3, 6 or 12 months | Passive comparison:  Blank control;  Null | Post-intervention:  100.0% | HIV testing |

Abbreviations: WRAPP: Wyoming Rural AIDS Prevention Project; SOLVE: Socially Optimized Learning in Virtual Environments; TMUS: Testing Makes Us Stronger; HMP: HealthMpowerment.org; HWUC: HIV Wake-Up Campaign; iPOL: Internet Popular Opinion Leaders; MiCHAT: Motivational Interviewing Communication about Health, Attitudes, and Thoughts; DMDN: Despre Mine. Despre Noi. (About Me. About Us.); CISS: Computer-Assisted Intervention for Safer Sex; MINTS: Men’s INTernet Study; HOPE: Harnessing Online Peer Education.; KIU: Keep It Up; CyBER/testing: Cyber-Based Education and Referral/testing; SD: Standard deviation ; NR: not reported; NA: not applicable; BIS: bisexual male; HES: heterosexual male; HOS: homosexual male; IGPP: independent-group pre-posttest design; RCT: randomized controlled trial; INT: intervention condition; COM: comparison condition; NRCT: non/quasi-randomized controlled trial; SGPP: single-group pre-posttest design; IGOP: independent-group only-posttest design; CS: cross-sectional study; RCS: repeated cross-sectional study; STI: sexually transmitted infection; UAI: unprotected anal intercourse; MSP: multiple sex partnership

a. Data were obtained through personal communication with the study authors.

b. Descriptive statistics for age were estimated based on raw data reported in the original articles.

c. The minimum control condition of the original RCT was selected as the comparison condition for meta-analysis.

d. The ehealth treatment condition of the original RCT was selected as the intervention condition for meta-analysis; the pre-intervention status was used as the comparison condition accordingly.

e. Both ehealth treatment conditions of the original RCT was separately included as the intervention conditions for meta-analysis; the control group was proportionally split to form two comparison conditions.

f. Two ehealth treatment conditions of the original RCT were combined and used as the intervention condition; the pre-intervention status was used as the comparison condition accordingly.

**References**

1. Anand T, Nitpolprasert C, Jantarapakde J, Meksena R, Phomthong S, Phoseeta P, et al. Implementation and impact of a technology-based HIV riskreduction intervention among Thai men who have sex with men using 'Vialogues:' A randomized controlled trial. Journal of the International AIDS Society Conference: 22nd International AIDS Conference, AIDS. 2018;21(Supplement 6).

2. Bauermeister JA, Pingel ES, Jadwin-Cakmak L, Harper GW, Horvath K, Weiss G, et al. Acceptability and preliminary efficacy of a tailored online HIV/STI testing intervention for young men who have sex with men: the Get Connected! program. AIDS & Behavior. 2015;19(10):1860-74.

3. Bourne C, Knight V, Guy R, Wand H, Lu H, McNulty A. Short message service reminder intervention doubles sexually transmitted infection/HIV re-testing rates among men who have sex with men. Sexually Transmitted Infections. 2011;87(3):229-31.

4. Bowen A, Williams M, Daniel C, Clayton S. Internet based HIV prevention research targeting rural MSM: Feasibility, acceptability, and preliminary efficacy. Journal of Behavioral Medicine. 2008;31(6):463-77.

5. Daniel CM, Bowen A, Williams M, Clayton S, Ross L. Assessment of long-term efficacy of an internet delivered HIV risk reduction intervention for rural MSM. Annals of Behavioral Medicine. 2008;35:S105-S.

6. Carpenter KM, Stoner SA, Mikko AN, Dhanak LP, Parsons JT. Efficacy of a web-based intervention to reduce sexual risk in men who have sex with men. AIDS & Behavior. 2010;14(3):549-57.

7. Chiasson MA, Shaw FS, Humberstone M, Hirshfield S, Hartel D. Increased HIV disclosure three months after an online video intervention for men who have sex with men (MSM). AIDS Care. 2009;21(9):1081-9.

8. Christensen JL, Miller LC, Appleby PR, Corsbie-Massay C, Godoy CG, Marsella SC, et al. Reducing shame in a game that predicts HIV risk reduction for young adult MSM: a randomized trial delivered nationally over the Web. Journal of the International AIDS Society. 2013;16(3 Suppl 2):18716.

9. Christensen JL. When it's good to feel bad: How responses to virtual environments predict real-life sexual risk-reduction [M.A.]. Ann Arbor: University of Southern California; 2007.

10. Davidovich U, De Wit J, Stroebe W. Using the Internet to reduce risk of HIV-infection in steady relationships: A randomized controlled trial of a tailored intervention for gay men. Liaisons dangereuses: HIV risk behavior prevention in steady gay relationships Amsterdam: Roel & Uigeefprojecten. 2006:95-122.

11. Desai M, Burns F, Mercey D, Nardone A, Muniina P, Sharp T, et al. Active recall of men who have sex with men (MSM) for an HIV/STI testing: A feasible and effective strategy? HIV Medicine. 2014;15:109.

12. Fernandez MI, Hosek SG, Hotton AL, Gaylord SE, Hernandez N, Alfonso SV, et al. A Randomized Controlled Trial of POWER: An Internet-Based HIV Prevention Intervention for Black Bisexual Men. AIDS and behavior. 2016;20(9):1951-60.

13. Greene GJ, Madkins K, Andrews K, Dispenza J, Mustanski B. Implementation and Evaluation of the Keep It Up! Online HIV Prevention Intervention in a Community-Based Setting. AIDS Education & Prevention. 2016;28(3):231-45.

14. Habarta N, Boudewyns V, Badal H, Johnston J, Uhrig J, Green D, et al. CDC'S Testing Makes Us Stronger (TMUS) Campaign: Was Campaign Exposure Associated With HIV Testing Behavior Among Black Gay and Bisexual Men? AIDS Education & Prevention. 2017;29(3):228-40.

15. Hightow-Weidman LB, Pike E, Fowler B, Matthews DM, Kibe J, McCoy R, et al. HealthMpowerment.org: feasibility and acceptability of delivering an internet intervention to young Black men who have sex with men. AIDS Care. 2012;24(7):910-20.

16. Hilliam A, Fraser L, Turner L. HIV Wake-Up Campaign Evaluation. Scotland: NHS Health Scotland; 2011 Feb 2011.

17. Hirshfield S, Chiasson MA, Joseph H, Scheinmann R, Johnson WD, Remien RH, et al. An online randomized controlled trial evaluating HIV prevention digital media interventions for men who have sex with men. PLoS ONE [Electronic Resource]. 2012;7(10):e46252.

18. Kasatpibal N, Viseskul N, Srikantha W, Fongkaew W, Surapagdee N, Grimes RM. Effects of Internet-based instruction on HIV-prevention knowledge and practices among men who have sex with men. Nursing & Health Sciences. 2014;16(4):514-20.

19. Ko NY, Hsieh CH, Wang MC, Lee C, Chen CL, Chung AC, et al. Effects of Internet popular opinion leaders (iPOL) among Internet-using men who have sex with men. Journal of Medical Internet Research. 2013;15(2):e40.

20. Lau J, Lau M, Cheung A, Tsui H. A randomized controlled study to evaluate the efficacy of an internet-based intervention in reducing HIV risk behaviors among men who have sex with men in Hong Kong. AIDS Care. 2008;20(7):820-8.

21. Lau JT, Lee AL, Tse WS, Mo PK, Fong F, Wang Z, et al. A Randomized Control Trial for Evaluating Efficacies of Two Online Cognitive Interventions With and Without Fear-Appeal Imagery Approaches in Preventing Unprotected Anal Sex Among Chinese Men Who Have Sex with Men. AIDS & Behavior. 2016;20(9):1851-62.

22. Lelutiu-Weinberger C, Pachankis JE, Gamarel KE, Surace A, Golub SA, Parsons JT. Feasibility, Acceptability, and Preliminary Efficacy of a Live-Chat Social Media Intervention to Reduce HIV Risk Among Young Men Who Have Sex With Men. AIDS & Behavior. 2015;19(7):1214-27.

23. Lelutiu-Weinberger C, Manu M, Ionescu F, Dogaru B, Kovacs T, Dorobantescu C, et al. An mHealth Intervention to Improve Young Gay and Bisexual Men's Sexual, Behavioral, and Mental Health in a Structurally Stigmatizing National Context. Jmir Mhealth and Uhealth. 2018;6(11).

24. Mi G, Wu Z, Wang X, Shi CX, Yu F, Li T, et al. Effects of a Quasi-Randomized Web-Based Intervention on Risk Behaviors and Treatment Seeking Among HIV-Positive Men Who Have Sex With Men in Chengdu, China. Current HIV Research. 2015;13(6):490-6.

25. Mikolajczak J, van Breukelen G, Kok G, Hospers H. Evaluation of an online HIV-prevention intervention to promote HIV-testing among men who have sex with men: a randomised controlled trial. Netherlands Journal of Psychology. 2012;67(2):21-35.

26. Mikolajczak J, Kok G, Hospers HJ. Queermasters: Developing a theory- and evidence-based internet HIV-prevention intervention to promote HIV-testing among men who have sex with men (MSM). Appl Psychol-Int Rev. 2008;57(4):681-97.

27. Mimiaga MJ, Thomas B, Biello K, Johnson BE, Swaminathan S, Navakodi P, et al. A Pilot Randomized Controlled Trial of an Integrated In-person and Mobile Phone Delivered Counseling and Text Messaging Intervention to Reduce HIV Transmission Risk among Male Sex Workers in Chennai, India. AIDS & Behavior. 2017;21(11):3172-81.

28. Mustanski B, Garofalo R, Monahan C, Gratzer B, Andrews R. Feasibility, acceptability, and preliminary efficacy of an online HIV prevention program for diverse young men who have sex with men: the keep it up! intervention. AIDS & Behavior. 2013;17(9):2999-3012.

29. Mustanski B, Parsons JT, Sullivan PS, Madkins K, Rosenberg E, Swann G. Biomedical and Behavioral Outcomes of Keep It Up!: An eHealth HIV Prevention Program RCT. American Journal of Preventive Medicine. 2018.

30. Keep It Up! 2.0: A Comparison of Two Online HIV Intervention Programs for Young Men Who Have Sex With Men (KIU!) [Internet]. U.S. National Library of Medicine. 2018. Available from: https://www.clinicaltrials.gov/ct2/show/results/NCT01836445?term=01836445&rank=1&view=results.

31. Nostlinger C, Platteau T, Bogner J, Buyze J, Dec-Pietrowska J, Dias S, et al. Implementation and Operational Research: Computer-Assisted Intervention for Safer Sex in HIV-Positive Men Having Sex With Men: Findings of a European Randomized Multi-Center Trial. Journal of Acquired Immune Deficiency Syndromes: JAIDS. 2016;71(3):e63-72.

32. Patel VV, Rawat S, Lelutiu-Weinberger C, Dange A, Kamath C, Poojary R, et al. CHALO! A social media based peer-delivered intervention increases HIV testing in men who have sex with men in Mumbai, India: a randomized trial. Journal of the International Aids Society. 2016;19.

33. Patel VV, editor A social media based peer-delivered HIV prevention intervention for men who have sex with men in Mumbai, India: a randomized trial comparing two messaging approaches. 21st International AIDS Conference (AIDS 2016); 2016; Durban, South Africa.

34. Prati G, Mazzoni D, Cicognani E, Albanesi C, Zani B. Evaluating the persuasiveness of an HIV mass communication campaign using gain-framed messages and aimed at creating a superordinate identity. Health Communication. 2016;31(9):1097-104.

35. Read SJ, Miller LC, Appleby PR, Nwosu ME, Reynaldo S, Lauren A, et al. Socially optimized learning in a virtual environment: Reducing risky sexual behavior among men who have sex with men. Human communication research. 2006;32(1):1-34.

36. Noar SM, Black HG, Pierce LB. Efficacy of computer technology-based HIV prevention interventions: a meta-analysis. AIDS. 2009;23(1):107-15.

37. Reback CJ, Grant DL, Fletcher JB, Branson CM, Shoptaw S, Bowers JR, et al. Text messaging reduces HIV risk behaviors among methamphetamine-using men who have sex with men. AIDS and Behavior. 2012;16(7):1993-2002.

38. Reback CJ, Fletcher JB, Swendeman DA, Metzner M. Theory-Based Text-Messaging to Reduce Methamphetamine Use and HIV Sexual Risk Behaviors Among Men Who Have Sex with Men: Automated Unidirectional Delivery Outperforms Bidirectional Peer Interactive Delivery. AIDS and behavior. 2019;23(1):37-47.

39. Rhodes SD, Vissman AT, Stowers J, Miller C, McCoy TP, Hergenrather KC, et al. A CBPR partnership increases HIV testing among men who have sex with men (MSM): outcome findings from a pilot test of the CyBER/testing internet intervention. Health Education & Behavior. 2011;38(3):311-20.

40. Rhodes SD, McCoy TP, Tanner AE, Stowers J, Bachmann LH, Nguyen AL, et al. Using social media to increase HIV testing among gay and bisexual men, other men who have sex with men, and transgender persons: Outcomes from a randomized community trial. Clinical Infectious Diseases. 2016;62(11):1450-3.

41. Rosser B, Oakes J, Konstan J, Hooper S, Horvath KJ, Danilenko GP, et al. Reducing HIV risk behavior of men who have sex with men through persuasive computing: Results of the Men's INTernet Study-II. Aids. 2010;24(13):2099-107.

42. Schonnesson LN, Bowen AM, Williams ML. Project SMART: Preliminary results from a test of the efficacy of a Swedish internet-based HIV risk-reduction intervention for men who have sex with men. Archives of Sexual Behavior. 2016;45(6):1501-11.

43. Solorio R, Norton-Shelpuk P, Forehand M, Montano D, Stern J, Aguirre J, et al. Tu Amigo Pepe: Evaluation of a Multi-media Marketing Campaign that Targets Young Latino Immigrant MSM with HIV Testing Messages. AIDS & Behavior. 2016;20(9):1973-88.

44. Tang W, Wei C, Cao B, Wu D, Li KT, Lu H, et al. Crowdsourcing to expand HIV testing among men who have sex with men in China: A closed cohort stepped wedge cluster randomized controlled trial. PLoS Medicine. 2018;15 (8) (no pagination)(e1002645).

45. Uhrig JD, Lewis MA, Bann CM, Harris JL, Furberg RD, Coomes CM, et al. Addressing HIV knowledge, risk reduction, social support, and patient involvement using SMS: results of a proof-of-concept study. Journal of Health Communication. 2012;17 Suppl 1:128-45.

46. Wang ZX, Lau JTF, Ip M, Ho SPY, Mo PKH, Latkin C, et al. A Randomized Controlled Trial Evaluating Efficacy of Promoting a Home-Based HIV Self-Testing with Online Counseling on Increasing HIV Testing Among Men Who Have Sex with Men. Aids and Behavior. 2018;22(1):190-201.

47. Ybarra ML, Prescott TL, Phillips GL, Bull SS, Parsons JT, Mustanski B. Pilot RCT results of an mHealth HIV prevention program for sexual minority male adolescents. Pediatrics. 2017;140 (1) (no pagination)(e20162999).

48. Young SD, Cumberland WG, Nianogo R, Menacho LA, Galea JT, Coates T. The HOPE social media intervention for global HIV prevention in Peru: a cluster randomised controlled trial. The Lancet HIV. 2015;2(1):e27-32.

49. Zou H, Fairley CK, Guy R, Bilardi J, Bradshaw CS, Garland SM, et al. Automated, computer generated reminders and increased detection of gonorrhoea, chlamydia and syphilis in men who have sex with men. PLoS ONE [Electronic Resource]. 2013;8(4):e61972.
