# Supplementary material for: The Effectiveness of Electronic Health Interventions for Promoting HIV-Preventive Behaviors Among Men Who Have Sex With Men: Meta-Analysis Based on an Integrative Framework of Design and Implementation Features
Source: J Med Internet Res. 2020 May 25;22(5):e15977. doi: 10.2196/15977 (PMC7281149; doi:10.2196/15977)
Supplement: Multimedia Appendix 4 [file jmir_v22i5e15977_app4.docx]

Multimedia Appendix 4. Design and implementation features of included interventions.

| **Study** ^a^ | **Tailoring strategy** | | | **TCS score**  **(C1, C2, C3)** | **Navigation style** | **Treatment**  **Duration** | **Operation mode** | | **Modality** | | | | **Intervention**  **adherence** |
| --- | --- | --- | --- | --- | --- | --- | --- | --- | --- | --- | --- | --- | --- |
|  | **Feed-**  **back** | **Adapta-tion** | **Persona-lization** |  |  |  | **HCI** | **CMC** | **Static site** | **Interactive module** | **Messag-ing** | **Social media** |  |
| Anand, 2018 [1] | 0 | 0 | 0 | 0 (0, 0, 0) ^b^ | tunneled | > 3 months | 1 [2] | 1 [2] | 0 | 0 | 0 | 1 | 89.2% |
| Bauermeister, 2015 [3] | 1 | 1 | 1 | 8 (2,6,1) | self-paced | ≤ 1 month | 1 | 0 | 1 | 0 | 0 | 0 | 100.0% |
| Bourne, 2011 [4] | 0 | 0 | 0 | 0 (0,0,0) | tunneled | <1 day | 1 | 0 | 0 | 0 | 1 | 0 | 100.0% |
| Bowen, 2008 [5] | 1 | 0 | 1 | 10 (3,7,1) | tunneled | ≤ 1 month | 1 | 0 | 0 | 1 | 0 | 0 | 69.2% |
| Carpenter, 2010 [6] | 1 | 1 | 0 | 9 (3,6,1) | tunneled | ≤ 1 month | 1 | 0 | 0 | 1 | 0 | 0 | 81.8% |
| Chiasson, 2009 [7] | 0 | 0 | 0 | 2 (1,1,0) | self-paced | <1 day | 1 | 0 | 1 | 0 | 0 | 0 | 54.0% |
| Christensen, 2013 [8] | 1 | 0 | 1 | 8 (2,6,1) | tunneled | NR | 1 | 0 | 0 | 1 | 0 | 0 | 100.0% |
| Christensen, 2007 [9] | 1 | 0 | 0 | 4 (2,3,1) | tunneled | <1 day | 1 | 0 | 0 | 1 | 0 | 0 | 70.0% |
| Davidovich, 2006 [10] | 1 | 0 | 0 | 8 (2,6,1) | self-paced | <1 day | 1 | 0 | 1 | 0 | 0 | 0 | 85.9% |
| Desai, 2014 [11] | 0 | 0 | 0 | 0 (0,0,0) | tunneled | >1 -3 months | 1 | 0 | 0 | 0 | 1 | 0 | 100.0% |
| Fernandez, 2016 [12] | 1 | 1 | 1 | 10 (3,6,1) | tunneled | ≤ 1 month | 0 | 1 | 0 | 0 | 1 | 0 | 61.1% |
| Greene, 2016 [13] | 1  [14] | 0  [14] | 0  [14] | 10 (3,6,1)  [14] | tunneled | <1 day | 1 | 0 | 1 | 1 | 0 | 0 | 100.0% |
| Habarta, 2017 [15] | 0 | 0 | 0 | 3 (2,2,0) | self-paced | > 3 months | 1 | 1 | 1 | 0 | 0 | 1 | 43.2% |
| Hightow-Weidman, 2012 [16] | 1  [16] | 0  [16] | 1  [16] | 10 (3,6,1)  [16] | tunneled [16] | ≤ 1 month | 1 | 1 | 1 | 1 | 1 | 0 | 63.6% |
| Hilliam, 2011 [17] | 0 | 0 | 0 | 0 (0,0,0) | self-paced | > 3 months | 1 | 0 | 1 | 0 | 1 | 0 | 72.96% |
| Hirshfield, 2012 -A [18] | 0 | 0 | 0 | 7 (3,4,0) | self-paced | <1 day | 1 | 0 | 1 | 0 | 0 | 0 | 48.60% |
| Hirshfield, 2012 -B [18] | 0 | 0 | 0 | 0 (0,0,0) | self-paced | <1 day | 1 | 0 | 1 | 0 | 0 | 0 | 45.30% |
| Kasatpibal, 2014 [19] | 0 | 0 | 0 | 4 (1,3,0)  [20] | self-paced | > 3 months | 1 | 0 | 1 | 1 | 0 | 0 | 100% |
| Ko, 2013 [21] | 0 | 0 | 0 | 6 (2,4,0) | self-paced | > 3 months | 1 | 1 | 0 | 0 | 0 | 1 | 25.5% |
| Lau, 2008 [22] | 1 | 0 | 0 | 0 (0,0,0) | tunneled | > 3 months | 1 | 0 | 0 | 0 | 1 | 0 | 74.1% |
| Lau, 2016 -A [23] | 0 | 0 | 0 | 7 (3,4,0) | self-paced | <1 day | 1 | 0 | 1 | 0 | 0 | 0 | 100.0% |
| Lau, 2016 -B [23] | 0 | 0 | 0 | 8 (3,5,0) | tunneled ^b^ | <1 day | 1 | 0 | 1 | 0 | 0 | 0 | 100.0% |
| Lelutiu-Weinberger, 2015 [24] | 1  [25, 26] | 1  [25, 26] | 1 | 10 (3,7,1)  [25, 26] | tunneled | >1 -3 months | 0 | 1 | 0 | 0 | 1 | 0 | 46.3% |
| Lelutiu-Weinberger, 2018 [27] | 1  [25, 26] | 1  [25, 26] | 1  [24] | 10 (3,7,1)  [25, 26] | tunneled | >1 -3 months | 0 | 1 | 0 | 0 | 1 | 0 | 77.6% |
| Mi, 2015  [28] | 1 | 0 | 0 | 0 (,0,0,0) | self-paced | > 3 months | 1 | 1 | 1 | 1 | 1 | 1 | 88.3% |
| Mikolajczak, 2012[29] | 1  [30] | 1  [30] | 1  [30] | 9 (2,7,1)  [30] | tunneled [30] | NR | 1 | 0 | 1 | 1 | 0 | 0 | 73.3% |
| Mimiaga, 2017 [31] | 1  [32] | 1  [32] | 1 | 7 (2,5,1)  [32] | tunneled | >1 -3 months | 0 | 1 | 0 | 0 | 1 | 0 | 96.0% |
| Mustanski, 2013 [14] | 1 | 0 | 0 | 10 (3,6,1) | tunneled | ≤ 1 month | 1 | 0 | 1 | 1 | 0 | 0 | 96.0% |
| Mustanski, 2018 [33] | 1 | 0 | 0 | 10 (3,6,1) | tunneled | ≤ 1 month | 1 | 0 | 1 | 1 | 0 | 0 | 84.3% |
| Nöstlinger, 2016 [34] | 1  [35] | 0 | 1 | 10 (2,7,2)  [35] | tunneled | >1 -3 months | 1 | 0 | 1 | 1 | 0 | 0 | 78.2% [35] |
| Patel, 2016 [36] | 0 | 0 | 0 | 3 (2,1,0)  [37] | tunneled | >1 -3 months | 1  [37] | 0  [37] | 0  [37] | 0  [37] | 1  [37] | 0  [37] | 90.2% |
| Prati, 2016 [38] | 0 | 0 | 0 | 7 (2,5,0) | self-paced | ≤ 1 month | 1 | 0 | 1 | 0 | 0 | 0 | 67.0% |
| Read, 2006 [39] | 1 | 0 | 0 | 8 (2,6,1) | tunneled | <1 day | 1 | 0 | 0 | 1 | 0 | 0 | 100.0% |
| Reback, 2012 [40] | 1  [41] | 1 | 1 | 6 (2,5,0)  [41] | tunneled | ≤ 1 month | 0 | 1 | 0 | 0 | 1 | 0 | 96.2% |
| Reback, 2019 [42] | 1 | 1 | 1 | 6 (2,5,0)  [40] | tunneled | >1 -3 months | 1 | 1 | 0 | 0 | 1 | 0 | 80.9% |
| Rhodes, 2011 [43] | 1  [44] | 1  [44] | 0 | 6 (2,5,0)  [44] | tunneled | > 3 months | 0 | 1 | 0 | 0 | 0 | 1 | 62.5% |
| Rhodes,  2016 [45] | 1  [43] | 1  [43] | 1  [43] | 7 (2,6,1)  [43] | tunneled | > 3 months | 0 | 1 | 0 | 0 | 1 | 0 | 87.6% |
| Rosser, 2010 [46] | 0 | 0 | 1 | 4 (3,2,0)  [47] | self-paced | ≤ 1 month | 1 | 0 | 1 | 1 | 0 | 0 | 81.9% |
| Schonnesson, 2016 [48] | 1  [5] | 0  [5] | 1  [5] | 10 (3,6,1)  [5] | tunneled | ≤ 1 month | 1 | 0 | 0 | 1 | 0 | 0 | 55.2% |
| Solorio, 2016 [49] | 0 | 0 | 0 | 6 (3,4,0) | self-paced | > 3 months | 1 | 0 | 1 | 1 | 1 | 0 | 97.7% |
| Tang, 2018 [50] | 0 | 0 | 0 | 0 (0,0,0) | tunneled | >1 -3 months | 1 | 1 | 0 | 0 | 1 | 1 | 93.3% |
| Uhrig, 2012 [51] | 0 | 1 | 0 | 3 (2,1,0)  [52] | tunneled | >1 -3 months | 0 | 1 | 0 | 0 | 1 | 0 | 76.9% |
| Wang, 2018 [53] | 0 | 1 | 1 | 7 (3,5,0) | tunneled | ≤ 1 month ^b^ | 0 | 1 | 1 | 0 | 1 | 0 | 81.8% |
| Ybarra, 2017 [54] | 1  [55] | 1  [55] | 0  [55] | 8 (2,6,1) | tunneled | >1 -3 months | 1  [55] | 1  [55] | 0 | 0 | 1 | 0 | 100.0% |
| Young, 2015 [56] | 1 | 0 | 0 | 2 (1,1,0) | tunneled | >1 -3 months | 0 | 1 | 0 | 0 | 0 | 1 | 90.7% |
| Zou, 2013 [57] | 0 | 0 | 0 | 0 (0,0,0) | tunneled | > 3 months | 1 | 0 | 0 | 0 | 1 | 0 | 100.0% |

Abbreviations and symbols: TCS= Theory Coding Scheme; HCI= Human-Computer Interaction; CMC= Computer-Mediated Communication. C1, C2 and C3 denotes the total scores of the TCS items categorized to “reference to underpinning theory”, “targeting of relevant theoretical constructs”, and “using theory to select recipients or tailor interventions”, respectively.

a. The relative coding rule was followed: the presence of unique characteristics in the intervention condition was coded.

b. Data were obtained through personal communications with the authors.

**References**

1. Anand T, Nitpolprasert C, Jantarapakde J, Meksena R, Phomthong S, Phoseeta P, et al. Implementation and impact of a technology-based HIV riskreduction intervention among Thai men who have sex with men using 'Vialogues:' A randomized controlled trial. Journal of the International AIDS Society Conference: 22nd International AIDS Conference, AIDS. 2018;21(Supplement 6).

2. Nitpolprasert C, editor Implementation and impact of a technology-based HIV risk-reduction intervention among Thai men who have sex with men using 'Vialogues': A randomized controlled trial. 22nd International AIDS Conference (AIDS 2018); 2018; Amsterdam, The Netherlands.

3. Bauermeister JA, Pingel ES, Jadwin-Cakmak L, Harper GW, Horvath K, Weiss G, et al. Acceptability and preliminary efficacy of a tailored online HIV/STI testing intervention for young men who have sex with men: the Get Connected! program. AIDS & Behavior. 2015;19(10):1860-74.

4. Bourne C, Knight V, Guy R, Wand H, Lu H, McNulty A. Short message service reminder intervention doubles sexually transmitted infection/HIV re-testing rates among men who have sex with men. Sexually Transmitted Infections. 2011;87(3):229-31.

5. Bowen A, Williams M, Daniel C, Clayton S. Internet based HIV prevention research targeting rural MSM: Feasibility, acceptability, and preliminary efficacy. Journal of Behavioral Medicine. 2008;31(6):463-77.

6. Carpenter KM, Stoner SA, Mikko AN, Dhanak LP, Parsons JT. Efficacy of a web-based intervention to reduce sexual risk in men who have sex with men. AIDS & Behavior. 2010;14(3):549-57.

7. Chiasson MA, Shaw FS, Humberstone M, Hirshfield S, Hartel D. Increased HIV disclosure three months after an online video intervention for men who have sex with men (MSM). AIDS Care. 2009;21(9):1081-9.

8. Christensen JL, Miller LC, Appleby PR, Corsbie-Massay C, Godoy CG, Marsella SC, et al. Reducing shame in a game that predicts HIV risk reduction for young adult MSM: a randomized trial delivered nationally over the Web. Journal of the International AIDS Society. 2013;16(3 Suppl 2):18716.

9. Christensen JL. When it's good to feel bad: How responses to virtual environments predict real-life sexual risk-reduction [M.A.]. Ann Arbor: University of Southern California; 2007.

10. Davidovich U, De Wit J, Stroebe W. Using the Internet to reduce risk of HIV-infection in steady relationships: A randomized controlled trial of a tailored intervention for gay men. Liaisons dangereuses: HIV risk behavior prevention in steady gay relationships Amsterdam: Roel & Uigeefprojecten. 2006:95-122.

11. Desai M, Burns F, Mercey D, Nardone A, Muniina P, Sharp T, et al. Active recall of men who have sex with men (MSM) for an HIV/STI testing: A feasible and effective strategy? HIV Medicine. 2014;15:109.

12. Fernandez MI, Hosek SG, Hotton AL, Gaylord SE, Hernandez N, Alfonso SV, et al. A Randomized Controlled Trial of POWER: An Internet-Based HIV Prevention Intervention for Black Bisexual Men. AIDS and behavior. 2016;20(9):1951-60.

13. Greene GJ, Madkins K, Andrews K, Dispenza J, Mustanski B. Implementation and Evaluation of the Keep It Up! Online HIV Prevention Intervention in a Community-Based Setting. AIDS Education & Prevention. 2016;28(3):231-45.

14. Mustanski B, Garofalo R, Monahan C, Gratzer B, Andrews R. Feasibility, acceptability, and preliminary efficacy of an online HIV prevention program for diverse young men who have sex with men: the keep it up! intervention. AIDS & Behavior. 2013;17(9):2999-3012.

15. Habarta N, Boudewyns V, Badal H, Johnston J, Uhrig J, Green D, et al. CDC'S Testing Makes Us Stronger (TMUS) Campaign: Was Campaign Exposure Associated With HIV Testing Behavior Among Black Gay and Bisexual Men? AIDS Education & Prevention. 2017;29(3):228-40.

16. Hightow-Weidman LB, Pike E, Fowler B, Matthews DM, Kibe J, McCoy R, et al. HealthMpowerment.org: feasibility and acceptability of delivering an internet intervention to young Black men who have sex with men. AIDS Care. 2012;24(7):910-20.

17. Hilliam A, Fraser L, Turner L. HIV Wake-Up Campaign Evaluation. Scotland: NHS Health Scotland; 2011 Feb 2011.

18. Hirshfield S, Chiasson MA, Joseph H, Scheinmann R, Johnson WD, Remien RH, et al. An online randomized controlled trial evaluating HIV prevention digital media interventions for men who have sex with men. PLoS ONE [Electronic Resource]. 2012;7(10):e46252.

19. Kasatpibal N, Viseskul N, Srikantha W, Fongkaew W, Surapagdee N, Grimes RM. Effects of Internet-based instruction on HIV-prevention knowledge and practices among men who have sex with men. Nursing & Health Sciences. 2014;16(4):514-20.

20. Kasatpibal N, Viseskul N, Srikantha W, Fongkaew W, Surapagdee N, Grimes RM. Developing a web site for human immunodeficiency virus prevention in a middle income country: a pilot study from Thailand. Cyberpsychol Behav Soc Netw. 2012;15(10):560-3.

21. Ko NY, Hsieh CH, Wang MC, Lee C, Chen CL, Chung AC, et al. Effects of Internet popular opinion leaders (iPOL) among Internet-using men who have sex with men. Journal of Medical Internet Research. 2013;15(2):e40.

22. Lau J, Lau M, Cheung A, Tsui H. A randomized controlled study to evaluate the efficacy of an internet-based intervention in reducing HIV risk behaviors among men who have sex with men in Hong Kong. AIDS Care. 2008;20(7):820-8.

23. Lau JT, Lee AL, Tse WS, Mo PK, Fong F, Wang Z, et al. A Randomized Control Trial for Evaluating Efficacies of Two Online Cognitive Interventions With and Without Fear-Appeal Imagery Approaches in Preventing Unprotected Anal Sex Among Chinese Men Who Have Sex with Men. AIDS & Behavior. 2016;20(9):1851-62.

24. Lelutiu-Weinberger C, Pachankis JE, Gamarel KE, Surace A, Golub SA, Parsons JT. Feasibility, Acceptability, and Preliminary Efficacy of a Live-Chat Social Media Intervention to Reduce HIV Risk Among Young Men Who Have Sex With Men. AIDS & Behavior. 2015;19(7):1214-27.

25. Pachankis JE, Lelutiu-Weinberger C, Golub SA, Parsons JT. Developing an online health intervention for young gay and bisexual men. AIDS Behav. 2013;17(9):2986-98.

26. Parsons JT, Lelutiu-Weinberger C, Botsko M, Golub SA. A randomized controlled trial utilizing motivational interviewing to reduce HIV risk and drug use in young gay and bisexual men. J Consult Clin Psychol. 2014;82(1):9-18.

27. Lelutiu-Weinberger C, Manu M, Ionescu F, Dogaru B, Kovacs T, Dorobantescu C, et al. An mHealth Intervention to Improve Young Gay and Bisexual Men's Sexual, Behavioral, and Mental Health in a Structurally Stigmatizing National Context. Jmir Mhealth and Uhealth. 2018;6(11).

28. Mi G, Wu Z, Wang X, Shi CX, Yu F, Li T, et al. Effects of a Quasi-Randomized Web-Based Intervention on Risk Behaviors and Treatment Seeking Among HIV-Positive Men Who Have Sex With Men in Chengdu, China. Current HIV Research. 2015;13(6):490-6.

29. Mikolajczak J, van Breukelen G, Kok G, Hospers H. Evaluation of an online HIV-prevention intervention to promote HIV-testing among men who have sex with men: a randomised controlled trial. Netherlands Journal of Psychology. 2012;67(2):21-35.

30. Mikolajczak J, Kok G, Hospers HJ. Queermasters: Developing a theory- and evidence-based internet HIV-prevention intervention to promote HIV-testing among men who have sex with men (MSM). Appl Psychol-Int Rev. 2008;57(4):681-97.

31. Mimiaga MJ, Thomas B, Biello K, Johnson BE, Swaminathan S, Navakodi P, et al. A Pilot Randomized Controlled Trial of an Integrated In-person and Mobile Phone Delivered Counseling and Text Messaging Intervention to Reduce HIV Transmission Risk among Male Sex Workers in Chennai, India. AIDS & Behavior. 2017;21(11):3172-81.

32. Thomas B, Closson EF, Biello K, Menon S, Navakodi P, Dhanalakshmi A, et al. Development and Open Pilot Trial of an HIV-Prevention Intervention Integrating Mobile-Phone Technology for Male Sex Workers in Chennai, India. Arch Sex Behav. 2017;46(4):1035-46.

33. Mustanski B, Parsons JT, Sullivan PS, Madkins K, Rosenberg E, Swann G. Biomedical and Behavioral Outcomes of Keep It Up!: An eHealth HIV Prevention Program RCT. American Journal of Preventive Medicine. 2018.

34. Nostlinger C, Platteau T, Bogner J, Buyze J, Dec-Pietrowska J, Dias S, et al. Implementation and Operational Research: Computer-Assisted Intervention for Safer Sex in HIV-Positive Men Having Sex With Men: Findings of a European Randomized Multi-Center Trial. Journal of Acquired Immune Deficiency Syndromes: JAIDS. 2016;71(3):e63-72.

35. Nostlinger C, Borms R, Dec-Pietrowska J, Dias S, Rojas D, Platteau T, et al. Development of a theory-guided pan-European computer-assisted safer sex intervention. Health Promot Int. 2016;31(4):782-92.

36. Patel VV, Rawat S, Lelutiu-Weinberger C, Dange A, Kamath C, Poojary R, et al. CHALO! A social media based peer-delivered intervention increases HIV testing in men who have sex with men in Mumbai, India: a randomized trial. Journal of the International Aids Society. 2016;19.

37. Patel VV, editor A social media based peer-delivered HIV prevention intervention for men who have sex with men in Mumbai, India: a randomized trial comparing two messaging approaches. 21st International AIDS Conference (AIDS 2016); 2016; Durban, South Africa.

38. Prati G, Mazzoni D, Cicognani E, Albanesi C, Zani B. Evaluating the persuasiveness of an HIV mass communication campaign using gain-framed messages and aimed at creating a superordinate identity. Health Communication. 2016;31(9):1097-104.

39. Read SJ, Miller LC, Appleby PR, Nwosu ME, Reynaldo S, Lauren A, et al. Socially optimized learning in a virtual environment: Reducing risky sexual behavior among men who have sex with men. Human communication research. 2006;32(1):1-34.

40. Reback CJ, Grant DL, Fletcher JB, Branson CM, Shoptaw S, Bowers JR, et al. Text messaging reduces HIV risk behaviors among methamphetamine-using men who have sex with men. AIDS and Behavior. 2012;16(7):1993-2002.

41. Reback CJ, Ling D, Shoptaw S, Rohde J. Developing a Text Messaging Risk Reduction Intervention for Methamphetamine-Using MSM: Research Note. Open AIDS J. 2010;4:116-22.

42. Reback CJ, Fletcher JB, Swendeman DA, Metzner M. Theory-Based Text-Messaging to Reduce Methamphetamine Use and HIV Sexual Risk Behaviors Among Men Who Have Sex with Men: Automated Unidirectional Delivery Outperforms Bidirectional Peer Interactive Delivery. AIDS and behavior. 2019;23(1):37-47.

43. Rhodes SD, Vissman AT, Stowers J, Miller C, McCoy TP, Hergenrather KC, et al. A CBPR partnership increases HIV testing among men who have sex with men (MSM): outcome findings from a pilot test of the CyBER/testing internet intervention. Health Education & Behavior. 2011;38(3):311-20.

44. Rhodes SD, Hergenrather KC, Duncan J, Ramsey B, Yee LJ, Wilkin AMJPiCHPR, Education,, et al. Using community-based participatory research to develop a chat room-based HIV prevention intervention for gay men. 2007;1(2):175-84.

45. Rhodes SD, McCoy TP, Tanner AE, Stowers J, Bachmann LH, Nguyen AL, et al. Using social media to increase HIV testing among gay and bisexual men, other men who have sex with men, and transgender persons: Outcomes from a randomized community trial. Clinical Infectious Diseases. 2016;62(11):1450-3.

46. Rosser B, Oakes J, Konstan J, Hooper S, Horvath KJ, Danilenko GP, et al. Reducing HIV risk behavior of men who have sex with men through persuasive computing: Results of the Men's INTernet Study-II. Aids. 2010;24(13):2099-107.

47. Rosser BR, Bockting WO, Rugg DL, Robinson BB, Ross MW, Bauer GR, et al. A randomized controlled intervention trial of a sexual health approach to long-term HIV risk reduction for men who have sex with men: effects of the intervention on unsafe sexual behavior. AIDS Educ Prev. 2002;14(3 Suppl A):59-71.

48. Schonnesson LN, Bowen AM, Williams ML. Project SMART: Preliminary results from a test of the efficacy of a Swedish internet-based HIV risk-reduction intervention for men who have sex with men. Archives of Sexual Behavior. 2016;45(6):1501-11.

49. Solorio R, Norton-Shelpuk P, Forehand M, Montano D, Stern J, Aguirre J, et al. Tu Amigo Pepe: Evaluation of a Multi-media Marketing Campaign that Targets Young Latino Immigrant MSM with HIV Testing Messages. AIDS & Behavior. 2016;20(9):1973-88.

50. Tang W, Wei C, Cao B, Wu D, Li KT, Lu H, et al. Crowdsourcing to expand HIV testing among men who have sex with men in China: A closed cohort stepped wedge cluster randomized controlled trial. PLoS Medicine. 2018;15 (8) (no pagination)(e1002645).

51. Uhrig JD, Lewis MA, Bann CM, Harris JL, Furberg RD, Coomes CM, et al. Addressing HIV knowledge, risk reduction, social support, and patient involvement using SMS: results of a proof-of-concept study. Journal of Health Communication. 2012;17 Suppl 1:128-45.

52. Coomes CM, Lewis MA, Uhrig JD, Furberg RD, Harris JL, Bann CM. Beyond reminders: a conceptual framework for using short message service to promote prevention and improve healthcare quality and clinical outcomes for people living with HIV. Aids Care-Psychological and Socio-Medical Aspects of Aids/Hiv. 2012;24(3):348-57.

53. Wang ZX, Lau JTF, Ip M, Ho SPY, Mo PKH, Latkin C, et al. A Randomized Controlled Trial Evaluating Efficacy of Promoting a Home-Based HIV Self-Testing with Online Counseling on Increasing HIV Testing Among Men Who Have Sex with Men. Aids and Behavior. 2018;22(1):190-201.

54. Ybarra ML, Prescott TL, Phillips GL, Bull SS, Parsons JT, Mustanski B. Pilot RCT results of an mHealth HIV prevention program for sexual minority male adolescents. Pediatrics. 2017;140 (1) (no pagination)(e20162999).

55. Ybarra ML, Prescott TL, Philips GL, 2nd, Bull SS, Parsons JT, Mustanski B. Iteratively Developing an mHealth HIV Prevention Program for Sexual Minority Adolescent Men. AIDS Behav. 2016;20(6):1157-72.

56. Young SD, Cumberland WG, Nianogo R, Menacho LA, Galea JT, Coates T. The HOPE social media intervention for global HIV prevention in Peru: a cluster randomised controlled trial. The Lancet HIV. 2015;2(1):e27-32.

57. Zou H, Fairley CK, Guy R, Bilardi J, Bradshaw CS, Garland SM, et al. Automated, computer generated reminders and increased detection of gonorrhoea, chlamydia and syphilis in men who have sex with men. PLoS ONE [Electronic Resource]. 2013;8(4):e61972.
