# Supplementary material for: The Effectiveness of Electronic Health Interventions for Promoting HIV-Preventive Behaviors Among Men Who Have Sex With Men: Meta-Analysis Based on an Integrative Framework of Design and Implementation Features
Source: J Med Internet Res. 2020 May 25;22(5):e15977. doi: 10.2196/15977 (PMC7281149; doi:10.2196/15977)
Supplement: Multimedia Appendix 5 [file jmir_v22i5e15977_app5.docx]

Multimedia Appendix 5. Results of moderation analyses.

1. Outcome: Unprotected anal intercourse

| **Moderators** | **Concurrent comparison (n=19)** ^a^ | | | | | |  | **Pre-intervention comparison (n=14)** ^a^ | | | | | |
| --- | --- | --- | --- | --- | --- | --- | --- | --- | --- | --- | --- | --- | --- |
|  | **n** | **d_+_** | ***P*** | **Q_w_ (*P*)** | **I^2^** | **Q_b_ (*P*)** |  | **n** | **d_+_** | **p** | **Q_w_ (*P*)** | **I^2^** | **Q_b_ (*P*)** |
| **Theory use** |  |  |  |  |  | 0.01 (.94) |  |  |  |  |  |  | 0.15 (.70) |
| Yes | 15 | -0.11 | .007 | 19.49 (.15) | 28.2% |  |  | 13 | -0.31 | < .001 | 17.90 (.12) | 33.0% |  |
| No | 4 | -0.12 | .33 | 4.80 (.19) | 37.5% |  |  | 1 | -0.36 | \ | \ | \ |  |
| **TCS score** ^b^ | 15 | -0.02 ^d^ | .25 ^d^ | \ | \ | \ |  | 13 | 0.00 ^d^ | .92 ^d^ | \ | \ | \ |
| **Tailoring strategy use** |  |  |  |  |  | 1.74 (.19) |  |  |  |  |  |  | 1.22 (.27) |
| Yes | 15 | -0.13 | <.001 | 14.19 (.44) | 1.3% |  |  | 6 | -0.26 | < .001 | 2.79 (.73) | 0.0% |  |
| No | 4 | -0.02 | .76 | 5.47 (.14) | 45.2% |  |  | 8 | -0.36 | < .001 | 13.76 (.06) | 49.1% |  |
| **Tailoring strategy no.** | 19 | -0.08 ^d^ | .01 ^d^ | \ | \ | \ |  | 14 | 0.03 ^d^ | .37 ^d^ | \ | \ | \ |
| **Use of feedback** |  |  |  |  |  | 4.00 (.04) |  |  |  |  |  |  | 1.82 (.18) |
| Yes | 13 | -0.15 | <.001 | 11.78 (.46) | 0.0% |  |  | 5 | -0.25 | < .001 | 1.44 (.84) | 0.0% |  |
| No | 6 | -0.03 | .60 | 6.68 (.24) | 25.1% |  |  | 9 | -0.37 | < .001 | 14.54 (.07) | 45.0% |  |
| **Use of adaptation** |  |  |  |  |  | 1.24 (.26) |  |  |  |  |  |  | 0.81 (.37) |
| Yes | 4 | -0.20 | .07 | 6.80 (.08) | 55.9% |  |  | 4 | -0.26 | < .001 | 1.57 (.66) | 0.0% |  |
| No | 15 | -0.07 | .03 | 14.99 (.38) | 6.6% |  |  | 10 | -0.32 | < .001 | 15.86 (.07) | 43.3% |  |
| **Use of personalization** |  |  |  |  |  | 1.08 (.30) |  |  |  |  |  |  | 0.76 (.38) |
| Yes | 6 | -0.16 | .04 | 10.11 (.07) | 50.5% |  |  | 4 | -0.27 | < .001 | 1.04 (.79) | 0.0% |  |
| No | 13 | -0.07 | .07 | 13.00 (.37) | 7.7% |  |  | 10 | -0.34 | < .001 | 16.50 (.06) | 45.5% |  |
| **Navigation style** ^c^ |  |  |  |  |  | 7.23 (.01) |  |  |  |  |  |  | 4.23 (.04) |
| Self-paced | 6 | -0.00 | 0.95 | 3.43 (.63) | 0.0% |  |  | 6 | -0.41 | < .001 | 3.65 (.60) | 0.0% |  |
| Tunneled | 13 | -0.16 | <.001 | 13.44 (.34) | 10.7% |  |  | 8 | -0.25 | < .001 | 9.21 (.24) | 24.0% |  |
| **Treatment duration** ^c^ |  |  |  |  |  | 1.44 (.70) |  |  |  |  |  |  | 4.88 (.18) |
| Single session | 5 | -0.04 | .54 | 4.30 (.37) | 7.0% |  |  | 4 | -0.36 | < .001 | 5.23 (.16) | 42.6% |  |
| < 1 month | 6 | -0.11 | .03 | 3.35 (.65) | 0.0% |  |  | 3 | -0.28 | < .001 | 0.73 (.70) | 0.0% |  |
| 1-3 months | 3 | -0.26 | .16 | 7.19 (.03) | 72.2% |  |  | 4 | -0.17 | .09 | 4.22 (.24) | 28.8% |  |
| > 3 months | 4 | -0.10 | .37 | 5.45 (.14) | 44.9% |  |  | 3 | -0.45 | < .001 | 1.00 (.61) | 0.0% |  |
| **Operation mode** |  |  |  |  |  | 0.59 (.44) |  |  |  |  |  |  | 0.81 (.37) |
| HCI | 12 | -0.08 | .01 | 8.67 (.65) | 0.0% |  |  | 10 | -0.34 | < .001 | 15.86 (.07) | 43.3% |  |
| CMC/ Combined | 7 | -0.16 | .09 | 15.53 (.02) | 61.4% |  |  | 4 | -0.26 | < .001 | 1.57 (.66) | 0.0% |  |
| **Modality type** |  |  |  |  |  | 0.98 (.91) |  |  |  |  |  |  | 4.39 (.22) |
| Static site | 3 | -0.03 | .72 | 2.95 (.23) | 32.2% |  |  | 4 | -0.40 | < .001 | 2.63 (.45) | 0.0% |  |
| Interactive module | 4 | -0.12 | .04 | 0.76 (.86) | 0.0% |  |  | 1 | -0.42 | .02 | \ | \ |  |
| Messaging | 3 | -0.16 | .29 | 7.68 (.02) | 74.0% |  |  | 5 | -0.21 | .003 | 5.48 (.24) | 27.0% |  |
| Social media | 2 | -0.22 | .45 | 5.25 (.02) | 80.9% |  |  | \ ^e^ | \ | \ | \ | \ |  |
| Combined | 7 | -0.11 | .03 | 4.15 (.66) | 0.0% |  |  | 4 | -0.35 | < .001 | 4.52 (.21) | 33.6% |  |
| **Adherence rate** | 19 | -0.27 ^d^ | .03 ^d^ | \ | \ | \ |  | 14 | -0.06 ^d^ | .82 ^d^ | \ | \ | \ |
| **Adherence level (relative)** |  |  |  |  |  | 9.13 (.01) |  |  |  |  |  |  | 1.96 (.38) |
| Low | 6 | 0.02 | .67 | 0.70 (.98) | 0.0% |  |  | 5 | -0.34 | < .001 | 0.48 (0.98) | 0.0% |  |
| Medium | 9 | -0.22 | .001 | 12.26 (.14) | 34.8% |  |  | 3 | -0.16 | .21 | 3.75 (.15) | 46.6% |  |
| High | 4 | -0.10 | .07 | 1.84 (.60) | 0.0% |  |  | 6 | -0.37 | < .001 | 9.02 (.11) | 44.5% |  |

Abbreviations and symbols: TCS= Theory Coding Scheme; HCI= Human-Computer Interaction; CMC= Computer-Mediated Communication. Q_w_ denotes the degree of within-group heterogeneity; Q_b_ denotes the degree of between-group difference in the pooled effect sizes.

a. Studies with exclusion of outliers.

b. Studies that reported the use of any theory.

c. Certain number of studies were excluded when relevant data were not reported or not applicable for this moderator.

d. Statistics for the meta-regression analysis: regression coefficient beta and its *P* value.

e. No studies with a pre-intervention comparison used social media as the intervention modality.

2. Outcome: HIV testing

| **Moderators** ^a^ | **n** | **d_+_** | ***P*** | **Q_w_ (*P*)** | **I^2^** | **Q_b_ (*P*)** |
| --- | --- | --- | --- | --- | --- | --- |
| **Theory use** |  |  |  |  |  | 0.82 (.36) |
| Yes | 14 | 0.39 | < .001 | 26.43 (.01) | 50.8% |  |
| No | 6 | 0.27 | .03 | 21.34 (<.001) | 76.6% |  |
| **TCS score** ^b^ | 14 | 0.02 ^d^ | .46 ^d^ | \ | \ | \ |
| **Tailoring strategy use** |  |  |  |  |  | 1.81 (.18) |
| Yes | 7 | 0.45 | < .001 | 6.89 (.33) | 12.9% |  |
| No | 13 | 0.32 | < .001 | 46.15 (< .001) | 74.0% |  |
| **Tailoring strategy no.** | 20 | 0.07 | .15 | \ | \ | \ |
| **Use of feedback** |  |  |  |  |  | 1.81 (.18) |
| Yes | 7 | 0.45 | < .001 | 6.89 (.33) | 12.9% |  |
| No | 13 | 0.32 | < .001 | 46.15 (< .001) | 74.0% |  |
| **Use of adaptation** |  |  |  |  |  | 1.71 (.19) |
| Yes | 5 | 0.44 | < .001 | 4.00 (.40) | 0.1% |  |
| No | 15 | 0.35 | < .001 | 49.02 (< .001) | 71.4% |  |
| **Use of personalization** |  |  |  |  |  | 2.76 (.10) |
| Yes | 3 | 0.51 | < .001 | 0.70 (0.70) | 0.0% |  |
| No | 17 | 0.35 | < .001 | 51.39 (< .001) | 68.9% |  |
| **Navigation style** |  |  |  |  |  | 2.37 (.12) |
| Self-paced | 10 | 0.29 | .001 | 23.81 (.005) | 62.2% |  |
| Tunneled | 10 | 0.45 | < .001 | 19.61 (.02) | 54.1% |  |
| **Treatment duration** ^c^ |  |  |  |  |  | 16.97 (<.001) |
| Single session/ ≤1 month | 6 | 0.15 | .18 | 4.15 (.53) | 0.0% |  |
| >1 -3 months | 5 | 0.48 | < .001 | 3.75 (.44) | 0.0% |  |
| > 3 months | 9 | 0.45 | < .001 | 18.21 (.02) | 56.1% |  |
| **Operation mode** |  |  |  |  |  | 4.90 (.09) |
| HCI | 12 | 0.26 | .006 | 44.95 (< .001) | 75.5% |  |
| CMC | 4 | 0.45 | < .001 | 3.78 (.29) | 20.6% |  |
| Combined | 4 | 0.50 | < .001 | 2.46 (.48) | 0.0% |  |
| **Use of HCI** |  |  |  |  |  | 1.51 (.22) |
| Yes | 16 | 0.34 | < .001 | 49.26 (< .001) | 69.5% |  |
| No | 4 | 0.45 | < .001 | 3.78 (.29) | 20.6% |  |
| **Use of CMC** |  |  |  |  |  | 4.38 (.04) |
| Yes | 8 | 0.47 | < .001 | 6.73 (.46) | 0.0% |  |
| No | 12 | 0.26 | .006 | 44.95 (< .001) | 75.5% |  |
| **Modality type** |  |  |  |  |  | 12.79 (.005) |
| Static site | 5 | 0.15 | .02 | 3.95 (.41) | 0.0% |  |
| Messaging | 7 | 0.44 | < .001 | 14.78 (.02) | 59.4% |  |
| Social media | 3 | 0.48 | < .001 | 4.32 (.11) | 53.7% |  |
| Combined | 5 | 0.40 | < .001 | 4.45 (.35) | 10.1% |  |
| **Adherence rate** ^c^ | 19 | 0.34 ^d^ | .14 ^d^ | \ | \ | \ |
| **Adherence level (relative)** ^c^ |  |  |  |  |  | 7.28 (.03) |
| Low | 7 | 0.24 | .008 | 21.27 (.002) | 71.8% |  |
| Medium | 5 | 0.43 | < .001 | 5.44 (.24) | 26.5% |  |
| High | 7 | 0.54 | < .001 | 7.40 (.28) | 18.9% |  |

Abbreviations and symbols: TCS= Theory Coding Scheme; HCI= Human-Computer Interaction; CMC= Computer-Mediated Communication. Q_w_ denotes the degree of within-group heterogeneity; Q_b_ denotes the degree of between-group difference in the pooled effect sizes.

a. Studies with exclusion of outliers.

b. Studies that reported any theory use.

c. Certain number of studies were excluded when relevant data were not reported (applicable) for this moderator.

d. Statistics for the meta-regression analysis: regression coefficient β and its *P* value.
